# Antimicrobial resistance patterns and genetic elements associated with the antibiotic resistance of *Helicobacter pylori* strains from Shanghai

**DOI:** 10.1186/s13099-022-00488-y

**Published:** 2022-03-30

**Authors:** Yixin Liu, Su Wang, Feng Yang, Wenjing Chi, Li Ding, Tao Liu, Feng Zhu, Danian Ji, Jun Zhou, Yi Fang, Jinghao Zhang, Ping Xiang, Yanmei Zhang, Hu Zhao

**Affiliations:** 1 Department of Laboratory Medicine, Huadong Hospital, Fudan University, Shanghai, China; 2 Shanghai Key Laboratory of Clinical Geriatric Medicine, Shanghai, China; 3 Research Center on Aging and Medicine, Fudan University, Shanghai, China; 4 Department of Endoscopy, Huadong Hospital, Fudan University, Shanghai, China

**Keywords:** *Helicobacter pylori*, Antibiotic resistance, Outer membrane protein, Virulence, CRISPRs, Variation, Genetic characteristics

## Abstract

**Background:**

Shanghai, in east China, has one of the world’s highest burdens of *Helicobacter pylori* infection. While multidrug regimens can effectively eradicate *H. pylori*, the increasing prevalence of antibiotic resistance (AR) in *H. pylori* has been recognized by the WHO as ‘high priority’ for urgent need of new therapies. Moreover, the genetic characteristics of *H. pylori* AR in Shanghai is under-reported. The purpose of this study was to determine the resistance prevalence, re-substantiate resistance-conferring mutations, and investigate novel genetic elements associated with *H. pylori* AR.

**Results:**

We performed whole genome sequencing and antimicrobial susceptibility testing of 112 *H. pylori* strains isolated from gastric biopsy specimens from Shanghai patients with different gastric diseases. No strains were resistant to amoxicillin. Levofloxacin, metronidazole and clarithromycin resistance was observed in 39 (34.8%), 73 (65.2%) and 18 (16.1%) strains, respectively. There was no association between gastroscopy diagnosis and resistance phenotypes. We reported the presence or absence of several subsystem protein coding genes including *hopE*, *hofF*, *spaB*, *cagY* and *pflA*, and a combination of CRISPRs, which were potentially correlated with resistance phenotypes. The *H. pylori* strains were also annotated for 80 genome-wide AR genes (ARGs). A genome-wide ARG analysis was performed for the three antibiotics by correlating the phenotypes with the genetic variants, which identified the well-known intrinsic mutations conferring resistance to levofloxacin (N87T/I and/or D91G/Y mutations in *gyrA*), metronidazole (I38V mutation in *fdxB*), and clarithromycin (A2143G and/or A2142G mutations in 23S rRNA), and added 174 novel variations, including 23 non-synonymous SNPs and 48 frameshift Indels that were significantly enriched in either the antibiotic-resistant or antibiotic-susceptible bacterial populations. The variant-level linkage disequilibrium analysis highlighted variations in a protease Lon with strong co-occurring correlation with a series of resistance-associated variants.

**Conclusion:**

Our study revealed multidrug antibiotic resistance in *H. pylori* strains from Shanghai, which was characterized by high metronidazole and moderate levofloxacin resistance, and identified specific genomic characteristics in relation to *H. pylori* AR. Continued surveillance of *H. pylori* AR in Shanghai is warranted in order to establish appropriate eradication treatment regimens for this population.

**Supplementary Information:**

The online version contains supplementary material available at 10.1186/s13099-022-00488-y.

## Introduction

*Helicobacter pylori* colonizes the gastric mucosa of 50–70% of the world’s population and increases the risk of developing chronic gastritis, peptic ulcer, gastric cancer, and gastric mucosa-associated lymphoid tissue (MALT) lymphoma [1–3]. Currently, in China, the recommended quadruple regimens for *H. pylori* eradication therapy include a proton pump inhibitor (PPI) and bismuth, accompanied by two antibiotics, including clarithromycin (CLA), metronidazole (MTZ), levofloxacin (LEV), and amoxicillin (AMX) [4, 5], with the LEV-containing regimen recommended as an alternative for rescue therapy. Moreover, the choice of regimens should be based on the local *H. pylori* antibiotic resistance (AR) and history of personal medication. However, *H. pylori* exhibits a diverse genomic structure with a high mutation rate and, under selective antibiotic pressure, manifests growing AR, especially to MTZ and CLA [6]. The rates of resistance of *H. pylori* to MTZ, CLA, and LEV in China have previously been measured as 40–70%, 20–50% and 20–50%, respectively [7]. Increasing rates of resistance have also been reported in Europe and America [5, 8], and antibiotic-resistant *H. pylori* has been recognized by the World Health Organization (WHO) as a “high priority” bacterium for which new therapies are urgently needed [9]. Given the limited number of antibiotics that are appropriate for *H. pylori* eradication and the worldwide increase in AR, the need for new efforts to comprehensively understand the mechanisms underlying *H. pylori* resistance to existing antibiotics is significant.

The genomic diversity of *H. pylori* is crucial for the establishment of associations between genetic elements and AR. A well-known example of the intrinsic resistance mechanism of *H. pylori* has been elucidated, showing that the emergence of resistance occurs mainly due to mutations in antimicrobial target genes. In particular, the single-nucleotide mutations in the target genes of CLA (23S rRNA), MTZ (*rdxA*, *frxA* and *fdxB*), and LEV (*gyrA* and *gyrB*) enable *H. pylori* to evade the corresponding antibiotic activity by inhibiting bacterial protein synthesis, hampering intracellular reduction-activation of MTZ, and blocking bacterial DNA replication and transcription, respectively [10, 11]. In particular, for MTZ resistance, there are several relevant mutations in the genes specified, especially in *rdxA*, where insertions and deletions, as well as non-synonymous mutations, can occur to disrupt or hamper the function of the RdxA protein. Ciprofloxacin and levofloxacin resistance is caused primarily by non-synonymous mutations, leading to amino acid substitutions at positions 87 and 91 in the GyrA protein [10, 11]. However, AR in *H. pylori* is a complex and multifactorial problem. Several other possible mechanisms have been described, including high expression of outer membrane proteins (OMPs) that form a permeability barrier to reduce antibiotic uptake [12], loss of porins with narrow pore channels causing decreased uptake of extracellular antibiotics [13], increased efflux of antibiotics through efflux pump transporters [14, 15], passive tolerance to antimicrobials by biofilm formation [16], and sporadic mutations with synergistic effects in the loci of some important protein synthesis factors, such as translation initiation factor IF-2 and ribosomal protein L22 [17, 18]. Furthermore, many other whole-genome associations with AR have been established in recent years. For instance, a significant association has been found between the virulence factor dupA1 genotype and the A2147G CLA resistance mutation [19], and a correlation has been observed between resistant *H. pylori* strains and CagA-negative strains [20]. Additionally, in Gram-negative bacteria, most studies have suggested an inverse relationship of the clustered regularly interspaced short palindromic repeat (CRISPR) loci with antimicrobial resistance in *Escherichia coli*, *Acinetobacter baumannii*, and *Klebsiella pneumoniae* [21–24], although some inconsistent observations showed either a positive or negative correlation of CRISPR-Cas with AR genes (ARGs) in some clinically important species [25]. However, although *H. pylori* is indeed one of the few bacterial species constitutively competent for natural transformation and uptake extracellular DNA [26], little is known about the relationship between CRISPRs and AR and acquired resistance genes in *H. pylori*.

Recently, based on the tools and databases used in AR studies, a genome-wide ARG analysis provided unprecedented insights into the global distribution of potential AR determinants of many pathogenic bacteria [27], facilitating the discovery of the molecular bases of the association between AR and genetic diversity. The aim of this study was to determine the resistance prevalence, re-substantiate resistance-conferring mutations, and investigate novel genetic elements associated with AR by specific subsystem analysis (locus level) and genome-wide ARG analysis (variant level), within the 112 clinically isolated *H. pylori* strains from Shanghai.

## Materials and methods

### Patients and samples

A total of 112 *H. pylori* strains (*H. pylori*-Shi) from 112 individual patients were successfully isolated from 437 gastric biopsy specimens collected during endoscopy in patients with gastric diseases who resided in Shanghai. Patients who had received treatment for *H. pylori* with any antibiotics, H_2_ receptor blockers, or proton pump inhibitors within 4 weeks before the study were excluded. All the biopsy samples were identified as being positive for *H. pylori* by morphological analysis and immunohistochemistry. The 112 gastric biopsy samples positive for *H. pylori* included 19 chronic superficial gastritis (CSG) samples, 66 chronic atrophic gastritis (CAG) samples, 8 gastric ulcer (GU) samples, 18 duodenal ulcer (DU) samples, and 1 gastric carcinoma (GC) sample. The demographics and clinical characteristics (gastroscopy diagnosis) of the 112 patients are shown in Additional file 2: Table S1. This study was approved by the Ethics Committee for Human Studies of Fudan University Huadong Hospital, with written informed consent obtained from all subjects (Ethics Approval Number: 2020K080).

### *H. pylori* isolation and culture

During endoscopy, two biopsy samples were obtained from the antrum, the 2–3 cm area in the front of the pylorus, of each patient. One sample was shipped immediately to a pathology centre for *H. pylori* detection in Huadong Hospital. The other sample was stored at − 80 °C and kept on dry ice during transfer. To culture *H. pylori*, the samples were homogenized and inoculated onto commercial *H. pylori*-selective plates (Nissui Pharmaceutical Co. Ltd., Tokyo, Japan), followed by incubation for 3–7 days at 37 °C under microaerophilic conditions (5% O_2_, 10% CO_2_, and 85% N_2_). Small, transparent/translucent colonies typical of *H. pylori* (Additional file 1: Fig. S1) were subsequently subjected to Gram staining, the rapid urease test (RUT), antibiotic susceptibility testing, and genomic DNA extraction.

### Antibiotic susceptibility testing

The AR of all the *H. pylori* strains to MTZ, CLA, LEV, and AMX was determined by both an E-test (Autobio, China) and the agar dilution method according to the protocols of the Clinical and Laboratory Standards Institute (Wayne, PA, USA). Briefly, after three subcultures of the individual strains isolated, the culture suspension turbidity of *H. pylori* was adjusted with saline to a McFarland opacity standard of 2.0 (approximately 6.0 × 10^9^ CFU/ml), and the suspensions were inoculated onto a Mueller–Hinton II agar plate (Becton Dickinson, Sparks, MD, USA) supplemented with 10% horse blood. The four drug E-test strips were attached to the plate and incubated at 37 °C for 3–5 days under microaerophilic conditions. Agar dilution minimum inhibitory concentration (MIC) tests were performed by serial twofold dilution of MTZ, CLA, LEV, and AMX, with the concentration of each ranging from 0.0039 to 256 mg/L. All drugs were purchased from Wako Pure Chemical Industry (Osaka, Japan). *H. pylori* resistance phenotypes were determined following the guidelines of the Clinical and Laboratory Standards Institute (Wayne, PA, USA) [28]. The breakpoints used were MIC ≥ 1 µg/ml for CLA, MIC ≥ 8 µg/ml for MTZ, MIC ≥ 1 µg/ml for LEV, and MIC ≥ 2 µg/ml for AMX. *H. pylori* ATCC 43504 was used as the control strain. Based on the antibiotic susceptibility testing results, the *H. pylori* strains could be divided into different AR categories in two patterns: The resistance (R)- susceptibility (S) pattern (MTZ-S and -R, CLA-S and -R, LEV-S and -R) and nonoverlapping patterns (MTZ mono-R, CLA mono-R, LEV mono-R, MTZ and CLA dual-R, MTZ and LEV dual-R, CLA and LEV dual-R, multiple-drug resistant (MDR) and susceptible to four antibiotics).

### *H. pylori* genomic DNA extraction and whole-genome sequencing

*H. pylori* strains were inoculated on Columbia agar (OXOID, Thermo Fisher Scientific Inc., Waltham, MA, USA) medium containing 8% sterile defibrinated sheep blood under microaerophilic conditions at 37 °C for 3–5 days. After three subcultures of the individual strains isolated, the total genomic DNA of *H. pylori* was extracted by the cetyltrimethyl ammonium bromide (CTAB) method [29]. All procedures were performed following the manufacturer’s instructions.

Genome sequencing was performed using the whole-genome shotgun (WGS) strategy based on the Illumina MiSeq platform (Illumina, San Diego, CA, USA) by constructing paired-end DNA libraries. The DNA library (~ 400 base-pair insert size) for each strain was prepared using the TruSeq DNA Sample Preparation Kit (Illumina, CA, USA). Briefly, genomic DNA was randomly uniformly fragmented in the range of 300–500 bases by sonication (Diagenode Bioruptor UCD-200). Illumina adapters were ligated to each fragment. Quality control of the libraries was performed by an Agilent 2100 Bioanalyzer (Agilent Technologies, Palo Alto, CA, USA) with a DNA 1000 chip according to the manufacturer’s instructions. The libraries were sequenced on an Illumina MiSeq sequencer using the TruSeq DNA Sample Preparation Kit (Illumina) with a paired-end 2 × 251-bp sequencing mode and a sequencing depth of more than 400× in all the strains. The quality of the raw reads was checked using FastQC (http://www.bioinformatics.babraham.ac.uk/projects/fastqc). To obtain high-quality reads, adapter contamination was removed by AdapterRemoval (version 2.1.7) [30], and all reads were filtered on SOAPec (version 2.0) [31] with a Kmer of 17 to trim the low-quality bases with a Phred quality score (Q value) of less than 20. The filtered clean reads were assembled by A5-miseq (version 20160825) [32] into contigs and scaffolds. The draft genomes of the 112 *H. pylori*-Shi strains were submitted to the National Center for Biotechnology Information (NCBI). The accession number of each genome is shown in Additional file 3: Table S2.

### *H. pylori* protein-coding gene, rRNA and CRISPR prediction

The genomic functional elements predicted included coding genes (CDSs), noncoding RNA, and CRISPRs. Glimmer software (version 3.0) [33] was used for gene prediction of the whole-genome sequence. The self-training gene prediction model was selected to extract the longest sequence in the assembled sequences, which was used for training the gene prediction model. Then, the gene prediction model constructed from this sequence was used for prediction of all gene sequences; the length of the open reading frame (ORF) was set to be no less than 110 bp, and the remaining parameters were the default settings of Glimmer 3.0. tRNA genes were identified by tRNAscan-SE (version 1.3.1) [34], and rRNA genes were identified by RNAmmer (version 1.2) [35]. CRISPRs were identified by CRISPRfinder (http://crispr.i2bc.paris-saclay.fr/Server/), and the direct repeats (DRs) and spacers in the whole genome were predicted by the CRISPR recognition tool [36].

### Functional annotation of predicted protein-coding genes in *H. pylori*

Functional annotations of all the predicted protein-coding genes were obtained using the Refseq database (NCBI-NR) (https://www.ncbi.nlm.nih.gov/refseq/) and SwissProt database (http://www.gpmaw.com/index.html). Each ORF was thus run through the two databases via blastall [37] with an E value less than 1e−6, at least 30% sequence identity and 70% query length coverage. The functional descriptions of the best hits in the databases were assigned to the corresponding protein-coding genes [38].

Functional classifications by clusters of orthologous groups (COGs) of proteins were analysed against the eggNOG (evolutionary genealogy of genes: Nonsupervised Orthologous Groups) database (version 5.0) [39]. The unique genes of the indicated categories were annotated using this database via blastp with a minimum E-value of 1e−6 and at least 70% for both sequence identity and query length coverage. The database assigned the best hits to the corresponding protein-coding genes, which were then classified into corresponding COG classes. The genes containing more than one domain from different categories were classified as multiple-class genes, whereas those that did not have a suitable hit against the database were classified as hypothetical protein-coding genes.

### *H. pylori* genome-wide gene family analysis

The dataset of the predicted protein sequences with a minimum of 50 amino acids in all 112 *H. pylori* strains was used for further orthology analysis. An all-vs-all blastp analysis was performed on the dataset with an E value of 1e−10 and 70% protein coverage cut-off applied for the alignments. The orthologous gene clusters in the sequence alignment results were identified using OrthoMCL (version 2.0.8) [40], with the inflation (I) value set to 1.5.

### *H. pylori* genomic subsystem analysis

Virulence factor-associated genes in the 112 *H. pylori* strains were predicted by aligning amino acid sequences in the Virulence Factor Database (VFDB) (http://www.mgc.ac.cn/VFs/) [41], which is a comprehensive database of pathogenic bacterial virulence factors containing 532 confirmed virulence factors from the literature and 2599 virulence-related genes from 74 genera of pathogenic bacteria, including *Helicobacter*. Each protein sequence was thus run through the database via blastp v.2.6.0+ to identify virulence-associated genes with an E-value less than 1e−5, greater than 60% identity, 70% coverage, and less than 10% gap length. To retrieve the given OMP family genes and efflux pump system genes, blastx v.2.5.0+ was used with an E-value less than 1e−6 and greater than 90% identity.

### Phylogenetic analysis of *H. pylori* strains

Based on the whole-genome orthologue analysis of the gene families, the single-copy orthologous genes were selected for multiple sequence alignment and quality control using the MAFFT tool (http://mafft.cbrc.jp/alignment/software/) and Gblocks (version 0.91) (http://molevol.cmima.csic.es/castresana/Gblocks.html). The phylogenetic tree was constructed by the maximum likelihood method in phyml software (version 3.1). The reliability of the phylogenetic tree branches was validated using the bootstrapping method with 1000 replications.

### Identification of acquired antimicrobial resistance genes

The command line version of ResFinder (version 4.1) with default parameters (90% identity and 60% coverage) was used to identify acquired antimicrobial resistance genes where resistance was conferred by a complete gene [42]. The ResFinder database is composed of 1649 acquired ARGs from 15 antibiotic drug classes.

### Genome-wide ARG analysis in *H. pylori*

The genome-wide resistance genes of the 112 *H. pylori* strains were identified from databases and the literature (Additional file 4: Table S3). First, the AR genes in the genomes of the 112 *H. pylori* strains were identified using the Comprehensive Antibiotic Research Database (CARD) (http://arpcard.mcmaster.ca) via BLAST [43]. An E value of 1e−6, 60% identity, and 70% protein coverage cut-off were applied for the alignments. The database includes genes involved in antibiotic susceptibility and/or resistance. To specifically identify β-lactamase enzyme-encoding genes, the β-lactamase database (BLDB) (version 1.0.11) (http://bldb.eu/) [44], a newly updated manually curated database for β-lactamases, was used with an E value of 1e−6, 80% identity, and 70% protein coverage cut-off. Because no hits were obtained in the database, no β-lactamase genes were included among the final genome-wide resistance genes.

To more comprehensively identify resistance genes in the genome, we compiled a literature-supported list of genes whose variation, presence or absence was associated with AR in *H. pylori*, including antibiotic targets and other sporadically reported genes that were either known to confer AR to *H. pylori* or subsequently found to be associated with resistance in *H. pylori*.

### Mapping and variant detection

To identify genetic alterations resulting in single-nucleotide polymorphisms (SNPs) and insertions or deletions (Indels), the sequencing reads of each strain were mapped to the *H. pylori* 26695 reference genome (NC_000915.1) using the Burrows–Wheeler Alignment Tool (version 0.7.12-r1039) [45]. The alignment files were subjected to local realignment and deduplication using Picard (version 1.107). The accuracy of variant calling was improved using the Genome Analysis Toolkit (GATK) [46]. Variant SNPs and Indels were called from each alignment file using GATK. Variant filtering was carried out by removing variants with mapping quality (MQ) < 40, quality depth (QD) < 2, and haplotype score > 13. The variants were annotated using ANNOVAR (Wang et al., 2010) to determine nonsynonymous SNPs (nsSNPs) and frameshift Indels (fsIndels). All variants identified in this study were manually inspected using the Integrative Genomics Viewer (version 2.3.86) [47].

### Linkage disequilibrium among variants potentially associated with AR or antibiotic susceptibility

The linkage disequilibrium (LD) of SNPs/Indels potentially associated with MTZ, CLA, and LEV resistance and susceptibility (*P* < 0.05) was assessed by calculating r2 and D′ values to identify the cooccurring correlation strength between each pair of variant sites using plink (version 1.07) [48]. The value of D′ was between − 1 and 1; a D′ value of 0 represented the two variants in a state of complete linkage equilibrium, and a D′ value of 1 represented the two variants in a state of complete LD.

### Crystal structures and functional domains of genome-wide resistance gene-encoding proteins with resistance-associated variations

The crystal structures of the proteins encoded by the indicated genes were obtained from SWISS-MODEL (https://swissmodel.expasy.org/) [49]. The structural model with sequence identity greater than 30%, Global Model Quality Estimation (GMQE) score greater than 0.6, and QMEAN Z score between − 4 and 0 was selected from the alignment results. The detailed model evaluation is shown in Additional file 1: Fig. S2. The functional domains of the indicated genes were identified using Pfam 34.0 (http://pfam.xfam.org/) [50]. All the significantly matched entries for each gene sequence were retained, and all nonsignificantly matched entries were discarded.

### Statistical analysis

When comparing the mean gene numbers between groups, Student’s t test was used. When analysing the association of each individual genotype with AR, frequencies between groups were compared using Fisher’s exact tests, Chi-squared tests or correction for continuity according to the data type to obtain the *P* value or adjusted *P* value. The statistical analysis was performed via RStudio (R 4.0.2) or GraphPad Prism (version 8.0).

## Results

### Antibiotic susceptibility and general genomic features of the 112 *H. pylori*-Shi strains

A total of 112 *H. pylori* strains from patients with digestive diseases were categorized based on antibiotic susceptibility phenotypes. The patients’ mean age was 52.2 ± 14.8 (mean ± SD) years, and 54.5% (61/112) were male (Table 1). The prevalence of MTZ-resistance was the highest (65.2%), with 33.9% MTZ mono-resistance, followed by LEV-resistance (34.8%), with 4.5% LEV mono-resistance, and CLA-resistance (16.1%), with 2.7% CLA mono-resistance. No strain was resistant to AMX. Among dual-resistant strains, the majority (20.5%) were resistant to both MTZ and LEV. A total of eight strains were MDR strains that were simultaneously resistant to MTZ, CLA, and LEV, and 28 were susceptible to all four antibiotics (Table 1). Sex, age, and gastroscopy diagnosis were not significantly associated with resistance (*P* > 0.05).

**Table 1:** Demographic information and antibiotic resistance rates

Group	Total, n (%)	Antibiotic-Resistance Phenotypes, n (%)								
		Amoxicillin-R/mono-R	Metronidazole-R/mono-R	Clarithromycin-R/mono-R	Levofloxacin-R/mono-R	Metronidazole-clarithromycin dual-R	Metronidazole-levofloxacin dual-R	Clarithromycin-levofloxacin dual-R	MDR	Susceptible to four antibiotics
Gender										
Male	61 (54.5)	0 (0)/0 (0)	40 (65.6)/23 (37.7)	8 (13.1)/3 (4.9)	19 (31.1)/4 (6.6)	2 (3.3)	12 (19.7)	0 (0)	3 (4.9)	14 (23.0)
Female	51 (45.5)	0 (0)/0 (0)	33 (64.7)/15 (29.4)	10 (19.6)/0 (0)	20 (39.2)/1 (2.0)	2 (3.9)	11 (21.6)	3 (5.9)	5 (9.8)	14 (23.0)
Age										
19–29	8 (7.1)	0 (0)/0 (0)	5 (62.5)/2 (25.0)	2 (25.0)/1 (12.5)	3 (37.5)/0 (0)	0 (0)	2 (25.0)	0 (0)	1 (12.5)	2 (25.0)
30–39	18 (16.1)	0 (0)/0 (0)	8 (44.4)/7 (38.9)	3 (16.7)/2 (11.1)	3 (16.7)/3 (16.7)	1 (5.6)	0 (0)	0 (0)	0 (0)	5 (27.8)
40–49	17 (15.2)	0 (0)/0 (0)	12 (70.6)/6 (35.3)	4 (23.5)/0 (0)	6 (35.3)/0 (0)	1 (5.9)	3 (17.6)	1 (5.9)	2 (11.8)	4 (23.5)
50–59	29 (25.9)	0 (0)/0 (0)	20 (69.0)/10 (34.5)	4 (13.8)/0 (0)	9 (31.0)/0 (0)	2 (6.9)	7 (24.1)	1 (3.4)	1 (3.4)	8 (27.6)
60–69	27 (24.1)	0 (0)/0 (0)	20 (74.1)/11 (40.7)	2 (7.41)/0 (0)	11 (40.7)/1 (3.7)	0 (0)	8 (29.6)	1 (3.7)	1 (3.7)	5 (18.5)
70–79	13 (11.6)	0 (0)/0 (0)	6 (46.2)/2 (15.4)	2 (15.4)/0 (0)	6 (46.2)/1 (7.7)	0 (0)	2 (15.4)	0 (0)	2 (15.4)	4 (30.8)
80 <	2 (1.8)	0 (0)/0 (0)	2 (100)/0(0)	1 (50)/0(0)	1 (50)/0(0)	0 (0)	1 (50)	0 (0)	1 (50)	0 (0)
Gastroscopy diagnosis										
Chronic superficial gastritis	19 (17.0)	0 (0)/0 (0)	8(42.1)/5(26.3)	2(10.5)/1(5.3)	5(26.3)/2(10.5)	0(0)	2(10.5)	0(0)	1(5.3)	8(42.1)
Chronic atrophic gastritis	66 (58.9)	0 (0)/0 (0)	46(69.7)/20(30.3)	13(19.7)/0(0)	29(43.9)/3(4.5)	3(4.5)	16(24.2)	3(4.5)	7(10.6)	14(21.2)
Gastric/Duodenal ulcer	26 (23.2)	0 (0)/0 (0)	18(69.2)/12(46.2)	3(11.5)/2(7.7)	5(19.2)/0(0)	1(3.8)	5(19.2)	0 (0)	0 (0)	6(23.1)
Gastric cancer	1 (0.9)	0 (0)/0 (0)	1(100)/1(100)	0 (0)/0 (0)	0 (0)/0 (0)	0 (0)/0 (0)	0 (0)/0 (0)	0 (0)/0 (0)	0 (0)/0 (0)	0 (0)/0 (0)
Total	112	0 (0)/0 (0)	73 (65.2)/38 (33.9)	18 (16.1)/3 (2.7)	39 (34.8)/5 (4.5)	4 (3.6)	23 (20.5)	3 (2.7)	8 (7.1)	28 (25.0)

The total sequence length and numbers of contigs, ORFs, and guanine-cytosine (GC) content for each genome of the 112 *H. pylori* strains grouped by AR phenotypes are shown in Additional file 3: Table S2. On average, the total length of contigs in the 112 *H. pylori* isolate genomes was 1.60 billion bp, encoding 1511–1624 genes per genome. A low average GC content, 38.7%, was identified, and the chromosome sizes ranged from 1.52 to 1.69 Mb. All sequenced genomes harboured 36 tRNA genes and an average of two rRNA genes.

### Genome-wide gene family analysis and unique resistance genes of *H. pylori* strains

**Fig. 1:**
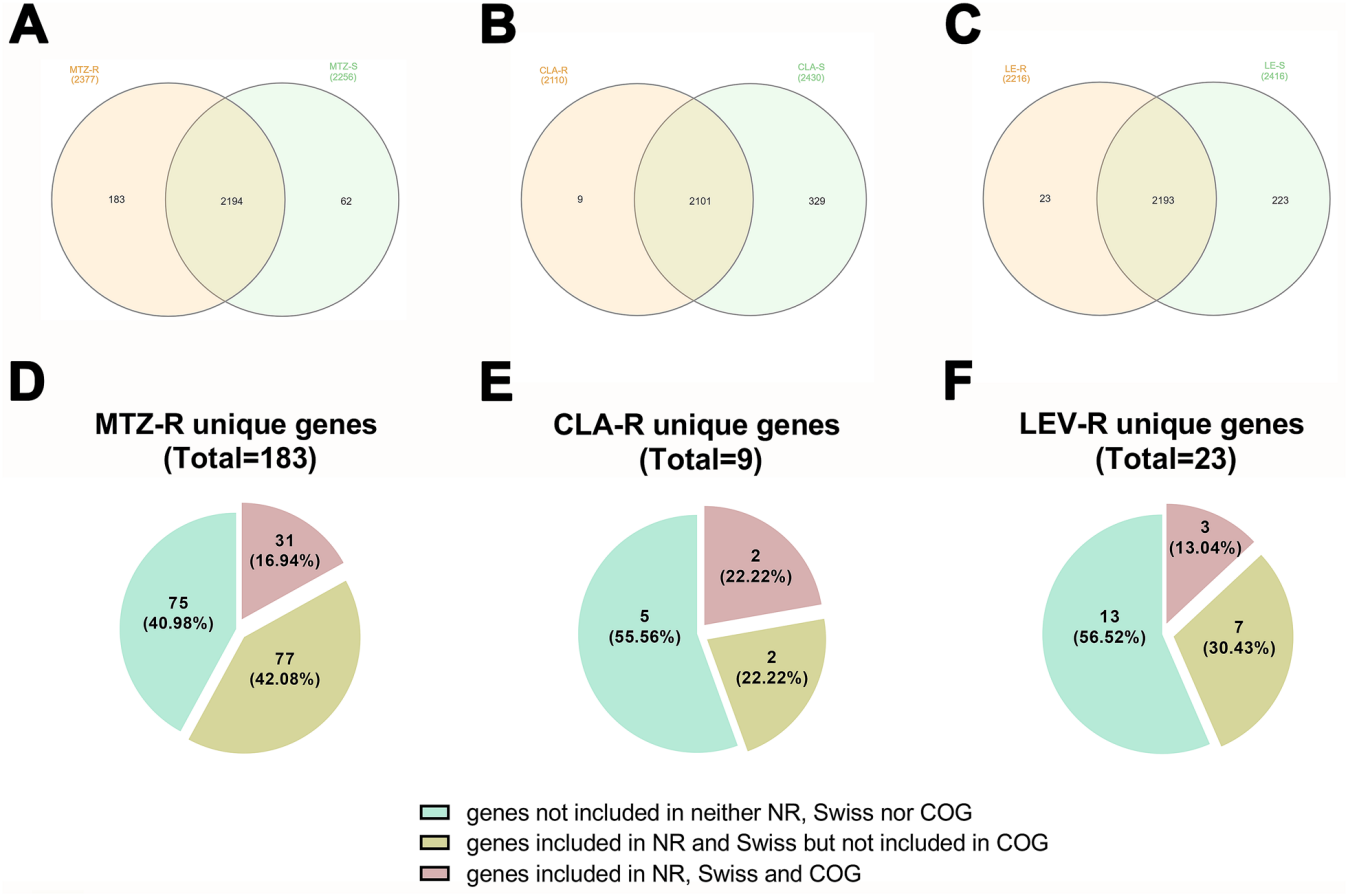
Genome-wide gene family analysis and functional classification of unique genes of antibiotic resistant categories. Venn diagrams showing the number of common and unique genes in MTZ- (**A**), CLA- (**B**) and LEV- (**C**) resistant (yellow) and susceptible (green) categories. The number in the parentheses indicates the total number of genes identified in each category. Pie diagrams revealing the status of unique genes of MTZ-R (**D**), CLA-R (**E**) and LEV-R (**F**) categories in NCBI-NR, SwissProt and COG databases

We initially conducted gene family analysis to define the shared genetic content of the resistant and susceptible categories and the unique gene pool for specific AR. Because different genomes possessed unique combinations of genes, the pan-genome size of the 112 genomes amounted to 2439, and the core-genome contains 1146 genes that were present in all genomes. A total of 2194 genes were shared between MTZ-S and MTZ-R, 2101 genes were shared between CLA-S and CLA-R, and 2193 genes were shared between LEV-S and LEV-R, accounting for 89.95%, 86.14% and 89.91% of the total genes, respectively. The shared genes between the corresponding resistant and susceptible categories here represented genes present in all and a subset of strains of both the resistant and susceptible categories but not those present in only the resistant or susceptible category. (Fig. 1A–C). Moreover, we identified 183, 9, and 23 unique genes in the MTZ, CLA, and LEV-R categories, respectively, including 75, 5, and 13 newly found genes that were not included in the NCBI-NR, SwissProt, or COG database (Fig. 1D–F, Additional file 5: Table S4). Among the remaining 108, 4 and 11 unique genes defined as unique resistance genes of the three AR categories (Additional file 6: Table S5), only 31, 2, and 3 genes could be assigned to various COG functional classes, respectively (Fig. 1D–F).

### Distributions of OMP family genes in resistant and susceptible categories

**Fig. 2:**
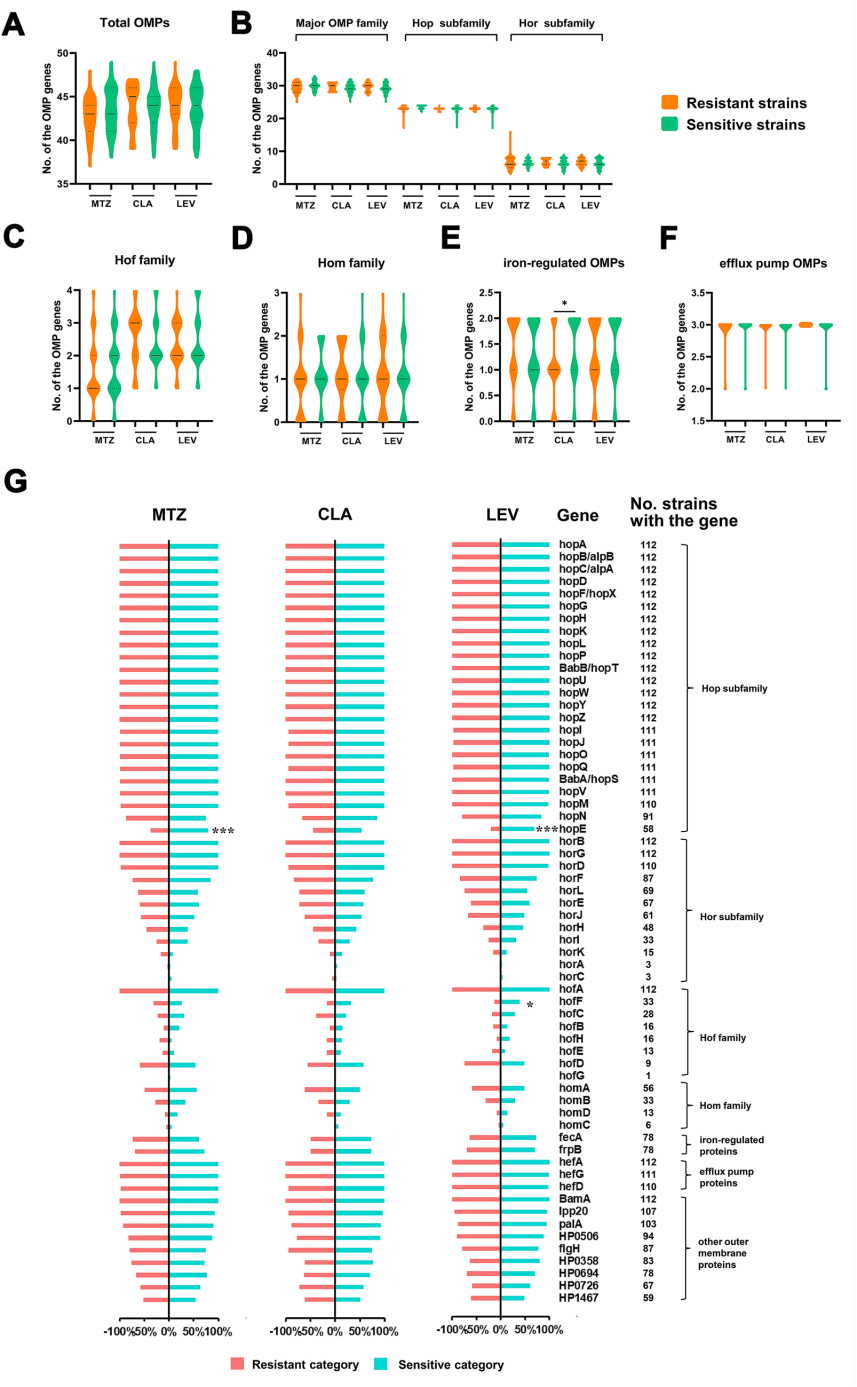
Analysis of OMP family genes in resistant and susceptible categories. Violin plots showing the distribution of the OMP genes in resistant and susceptible categories. The average presence levels of the total 62 OMP genes (**A**), the major OMP family genes along with two subfamilies genes (**B**), Hof family genes (**C**), Hom family genes (**D**), iron-regulated OMPs genes (**F**) and efflux pump OMP genes (**F**) in resistant and susceptible categories were compared, irrespective of the number of alleles of the genes. Solid lines indicate median levels and dotted lines indicate quartiles levels. **G** Proportion of strains with each OMP family gene in resistant and susceptible categories. Only genes with greater than 90% coverage were labelled as present. The number of strains containing the gene were listed on the right. **A**–**G** *P < 0.05, ***P < 0.001. Where there were no statistical analysis results labeled, there were no significant difference

To investigate whether OMP family genes were related to specific AR, the presence and absence of genes predicted to encode OMPs in different categories were analysed, including the currently identified five paralogous gene families of the major OMP family, including the Hop subfamily and Hop-related (Hor) subfamily, Hof family, Hom family, iron-regulated OMPs, and efflux pump OMPs, ranging in size from 3 to 36 members, as well as 9 other putative OMPs not in paralogous families (Additional file 7: Table S6). We first compared the average numbers of OMP genes in the resistant and susceptible categories for the three antibiotics irrespective of the number of alleles of the genes. There were no significant differences in the average number of total OMP genes, the major OMP family genes, Hof and Hom family genes, and efflux pump OMP genes (Fig. 2A–F). Intriguingly, we found that the average frequency of the iron-regulated OMP genes (*fecA* and *frpB*) in the CLA-R category was significantly lower than that in the CLA-S category (*P* = 0.0103). Notably, the frequencies of these genes in the strains varied greatly. Nearly all the strains contained the majority of the Hop subfamily genes (not including *hopE* and *hopN*), the efflux pump OMP genes *horB*, *horG*, *horD*, and *hofA* belonging to the hor or hof subfamily, and the gene encoding a β-barrel assembly machinery (BAM) component BamA and murein lipoprotein 20 (Lpp20) (Fig. 2G). However, the *horA*, *horC* and *hofG* genes were present in very few strains. Remarkably, the proportion of strains carrying *hopE* in both the MTZ- and LEV-S categories was significantly higher than that in the corresponding resistant category (Fig. 2G). Another gene with a significantly lower frequency in the resistant category was *hofF* (LEV).

### Distributions of virulence-associated genes in the resistant and susceptible categories

**Fig. 3:**
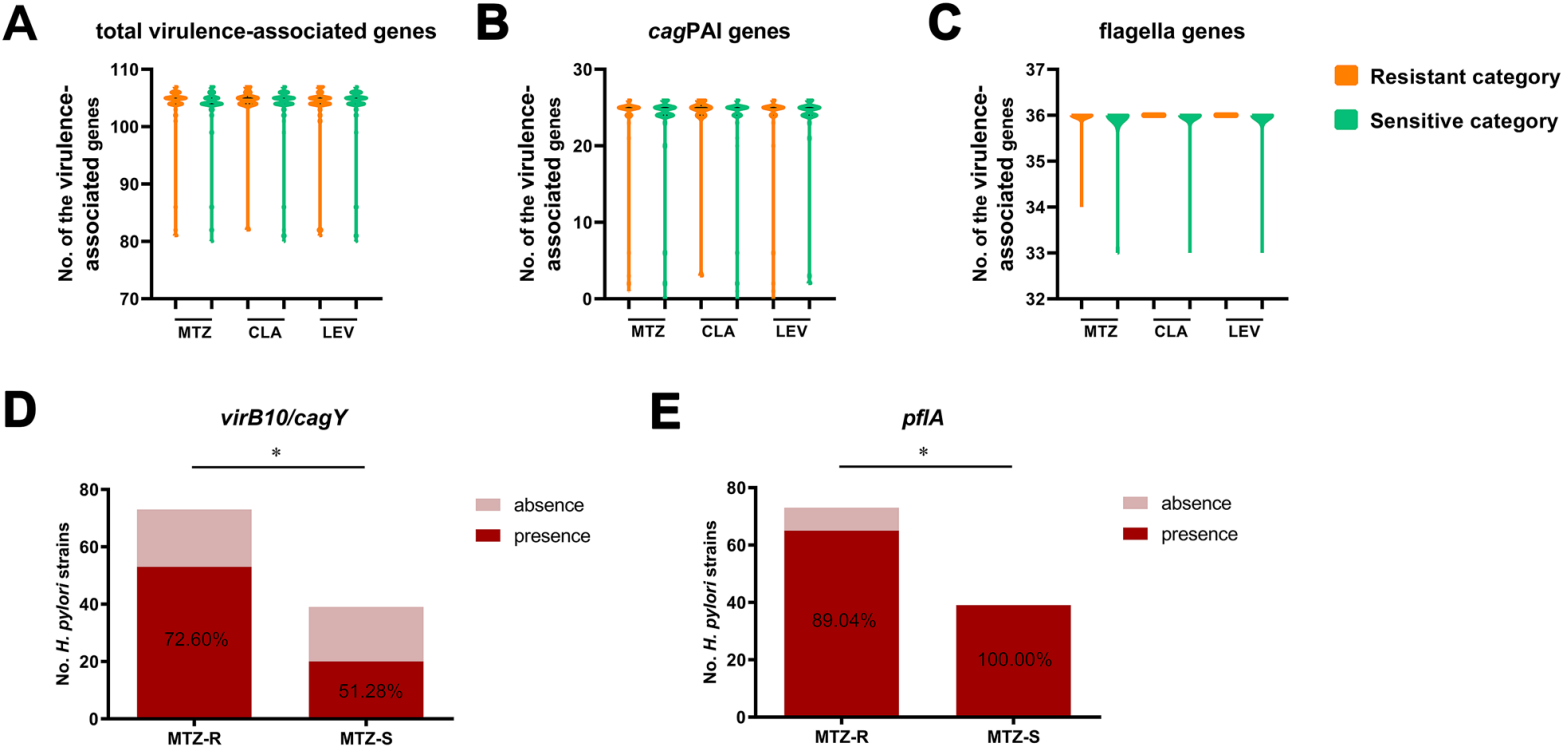
Analysis of virulence-associated genes in resistant and susceptible categories. Violin plots showing the distribution of the virulence-associated genes in resistant and susceptible categories. Comparisons of the average presence levels of the total virulence-associated genes (**A**), *cag*PAI genes (**B**) and flagellar genes (**C**) between resistant and susceptible categories were conducted, irrespective of the number of alleles of the genes. Solid lines indicate median levels and dotted lines indicate quartiles levels. Where there were no statistical analysis results labeled, there were no significant difference. **D** Two virulence-associated genes with significant different frequencies in MTZ-R and MTZ-S categories. *P < 0.05

To investigate whether the occurrence of virulence factor genes carried by *H. pylori* was related to specific AR, the presence and absence of 108 predicted virulence-associated genes against the VFDB in the resistant and susceptible categories were analysed, including 26 *cag* pathogenicity island (*cag*PAI) genes, 36 flagellar genes, and 47 other virulence-associated genes (Additional file 8: Table S7). Similarly, we compared the average numbers of virulence-associated genes in the resistant and susceptible categories irrespective of the number of alleles of the genes. There were no significant differences in the number of total virulence-associated genes, *cag*PAI genes, or flagellar genes (Fig. 3A–C). Additionally, the frequencies of these genes in the resistant and susceptible categories were compared, and there was no significant difference in the distribution of most of the genes (Additional file 6: Table S5). Notably, the frequencies of *cagY* (HP0527) and *pflA* (HP1274) were significantly higher (*P* = 0.0240) and lower (*P* = 0.0488), respectively, in the MTZ-R category than in the MTZ-S category (Fig. 3D, E).

### Distributions of the efflux pump genes in the resistant and susceptible categories

**Fig. 4:**
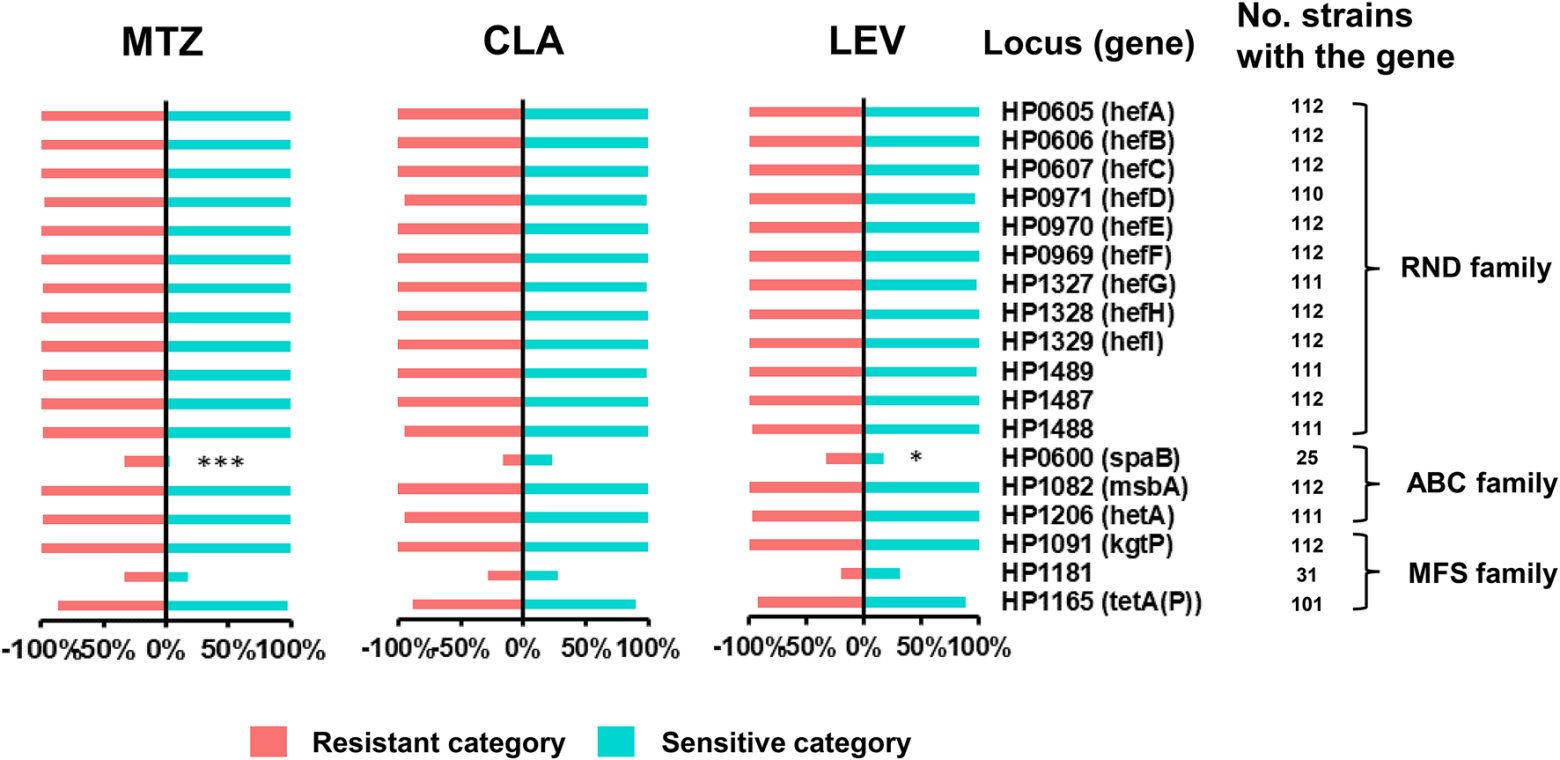
Analysis of efflux pump genes in resistant and susceptible categories. Proportion of strains with each efflux pump gene in resistant and susceptible categories. The number of strains containing the gene were listed on the right. *P < 0.05, ***P < 0.001. Where there were no statistical analysis results labeled, there were no significant difference

To investigate whether the efflux pump genes were related to specific AR, the presence and absence of 18 literature-based efflux pump genes were analysed, including 12 resistance-nodulation-cell division (RND) family genes, 3 ATP-binding cassette (ABC) family genes, and 3 major facilitator superfamily (MFS) family genes (Fig. 4). Among the 18 genes, 16 were present in almost all 112 *H. pylori* strains, and the remaining two genes, namely, *spaB* (HP0600) and HP1181, were absent in most of these strains, being detected in 25 and 31 strains, respectively. Nevertheless, the proportion of strains carrying *spaB* in the MTZ-R (*P* = 0.0002) and LEV-R (*P* = 0.0408) categories was obviously higher than that in the MTZ-S and LEV-S categories, respectively (Fig. 4).

### Correlation analysis of CRISPRs and their components with *H. pylori* AR

Given the natural function of the CRISPR-Cas system as an adaptive defence mechanism against foreign DNA and the controversial relationship between the CRISPR-Cas system and acquired ARGs in bacterial genomes [21–25], we conducted predictions for CRISPR-Cas systems and acquired ARGs where resistance was conferred by a complete gene rather than mutations, seeking to explore the relationship among CRISPR-Cas, acquired ARGs, and AR in *H. pylori*. Unfortunately, the results suggested that neither ARGs nor *cas* genes were found in the 112 *H. pylori* strains. However, a total of 153 CRISPR loci were predicted in 83 strains, ranging in length from 65 to 496 bp.

**Fig. 5:**
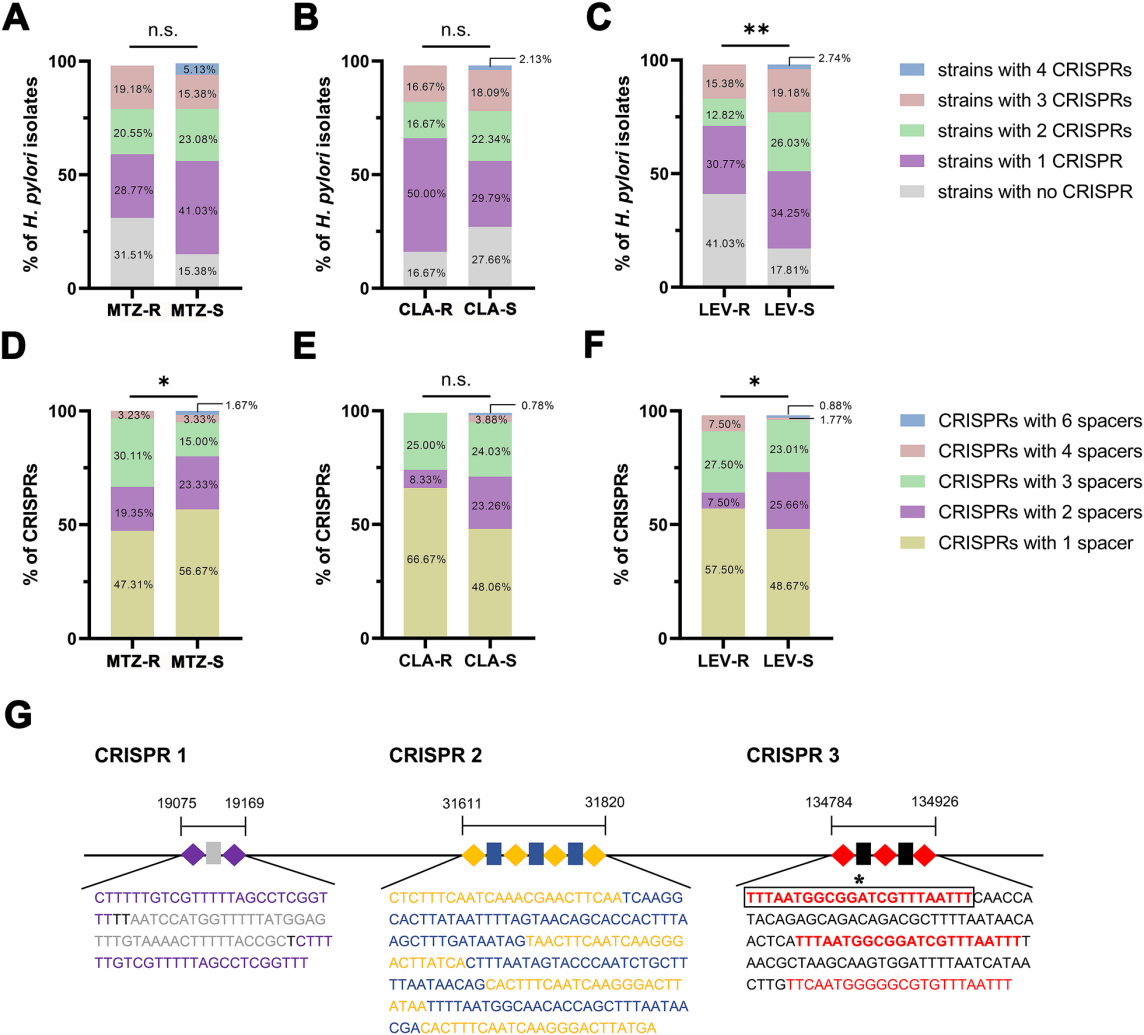
Distribution and constitution of CRISPRs in resistant and susceptible categories and a previously unknown CRISPRs combination present exclusively in seven MTZ mono-R strains**.** The proportions of strains with 0, 1, 2, 3 and 4 CRISPRs in resistant and susceptible categories of MTZ (**A**), CLA (**B**) and LEV (**C**), respectively. **P < 0.01, n.s. not significant. The proportions of CRISPRs with 1, 2, 3, 4 and 6 spacers in resistant and susceptible categories of MTZ (**D**), CLA (**E**) and LEV (**F**), respectively. *P < 0.05, n.s. not significant. **G** The structural analysis of a previously unknown CRISPRs combination presented exclusively in seven MTZ mono-R strains, including DRs (purple, yellow and red diamonds in each CRISPR) and spacers (grey, blue and black rectangles in each CRISPR). The base sequences are shown below the CRISPR array. The DRs and spacers in the colored characters correspond with the colors of the respective diamonds and rectangles. The DR sequence in box, which exclusively presented in nine MTZ-R strains, indicated that there was a significant difference of the DR frequencies between MTZ-R and MTZ-S categories. *P < 0.05

Because of the different CRISPR contents of the strains, the proportions of strains with 0, 1, 2, 3, and 4 CRISPRs in the resistant and susceptible categories of MTZ, CLA and LEV were analysed (Fig. 5A–C). Notably, the proportion of the strains with no CRISPR in the LEV-R category was significantly higher than that in the LEV-S category (*P* = 0.0075, Fig. 5C). Because of the different spacer contents of the CRISPRs, the proportion of CRISPRs with 1, 2, 3, 4, and 6 spacers was also analysed (Fig. 5D–F). Interestingly, the proportion of CRISPRs with three spacers in the MTZ-R category was significantly higher than that in the MTZ-S category (*P* = 0.0331, Fig. 5D), and the proportion of CRISPRs with two spacers in the LEV-R category was significantly lower than that in the LEV-S category (*P* = 0.0152, Fig. 5F). Similarly, the proportion of CRISPRs with two or three spacers in the resistant category of the other two antibiotics was also higher or lower, respectively, than that in the corresponding susceptible category, although there was no significant difference (Fig. 5D–F). In addition, detailed analysis of the DR and spacer sequences showed that a CRISPR combination containing the three most common CRISPRs in *H. pylori* coexisted in seven MTZ mono-R strains (Fig. 5G). Of note, one of the DRs in the CRISPR combination occurred in the CRISPRs of 9 strains, which included CRISPRs in seven MTZ mono-R strains and 2 CRISPRs (Additional file 1: Fig. S3) in two other MTZ-R strains (a MTZ mono-R strain and a MTZ and CLA dual-R strain), leading to a significantly higher proportion of strains with the DR in the MTZ-R category than in the MTZ-S category (*P* = 0.0259, Fig. 5G, Additional file 9: Table S8).

### Phylogenetic analysis and relationship of AR patterns and specific genetic loci with strain relatedness in *H. pylori* strains

**Fig. 6:**
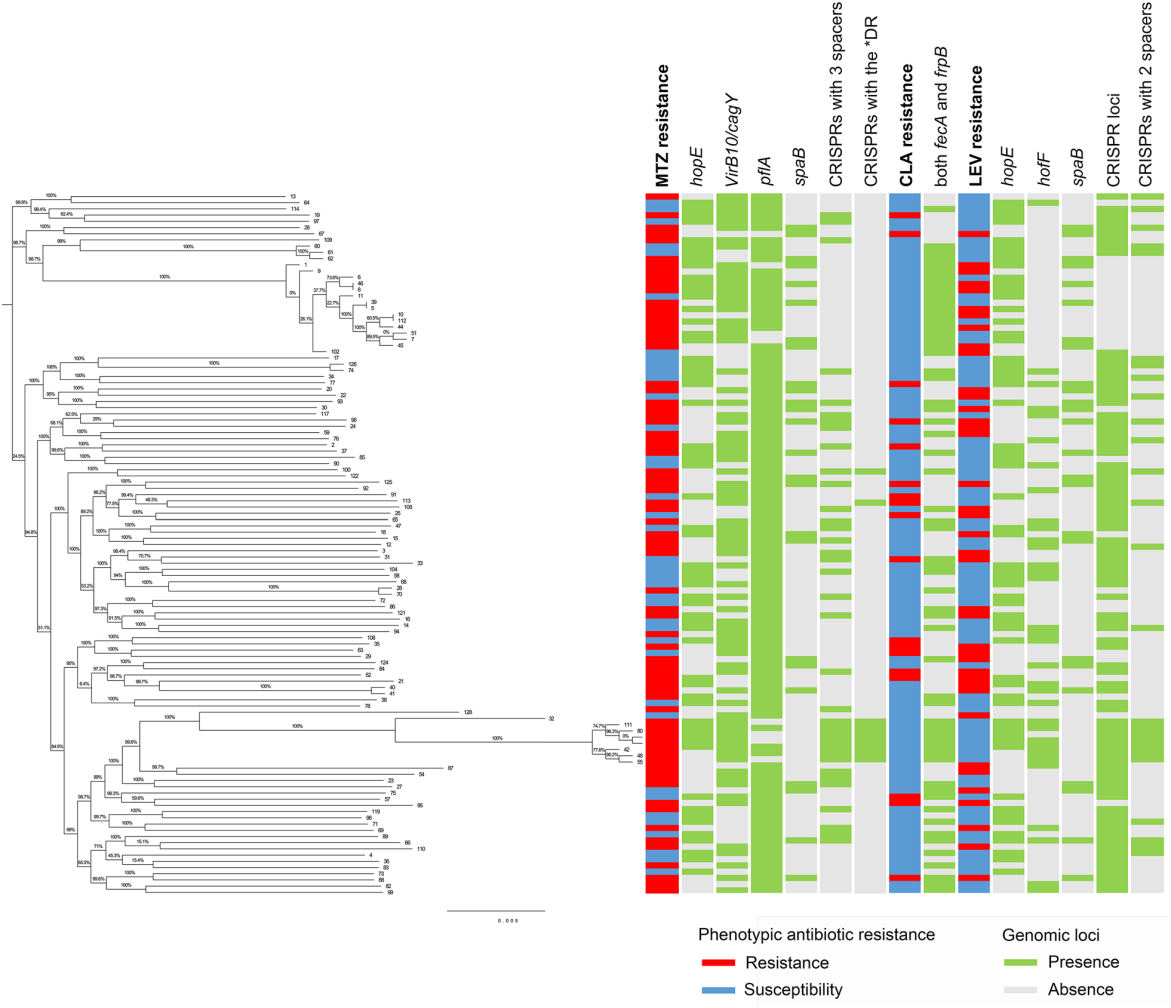
Phylogenetic analysis with respect to antibiotic resistance patterns and specific genetic loci profiles in *H. pylori* strains. Maximum likelihood phylogenetic tree based on whole-genome level single-copy genes of the 112 *H. pylori* strains, the resistance patterns, and the significantly different genomic loci observed above between susceptible and resistant categories for the corresponding antibiotic. The susceptible and resistant patterns are denoted by blue and red rectangles, respectively. The presence and absence of the specific loci are denoted by green and grey rectangles, respectively

When constructing the maximum likelihood tree of the 112 *H. pylori*-Shi genomes based on the whole-genome level single-copy genes with respect to different resistance patterns and the profile of the observed differentially abundant subsystem genomic loci between the susceptible and resistant categories for the corresponding antibiotic, no strain relatedness was found to be associated with either each single antibiotic phenotype or the presence/absence of the differentially abundant loci, including genes encoding OMPs (*hopE*, *hofF* and iron-regulated OMPs), virulence-associated genes (*virB10*/*cagY* and *pflA*), efflux pump genes (*spaB*), and CRISPRs with 2 or 3 spacers or the indicated DR sequences (Fig. 6). Alternatively, these strains had distinct susceptibility and associated locus profiles regardless of genetic relatedness among strains.

### Genome-wide genetic variations and prevalence of variations in *H. pylori* ARGs

**Fig. 7:**
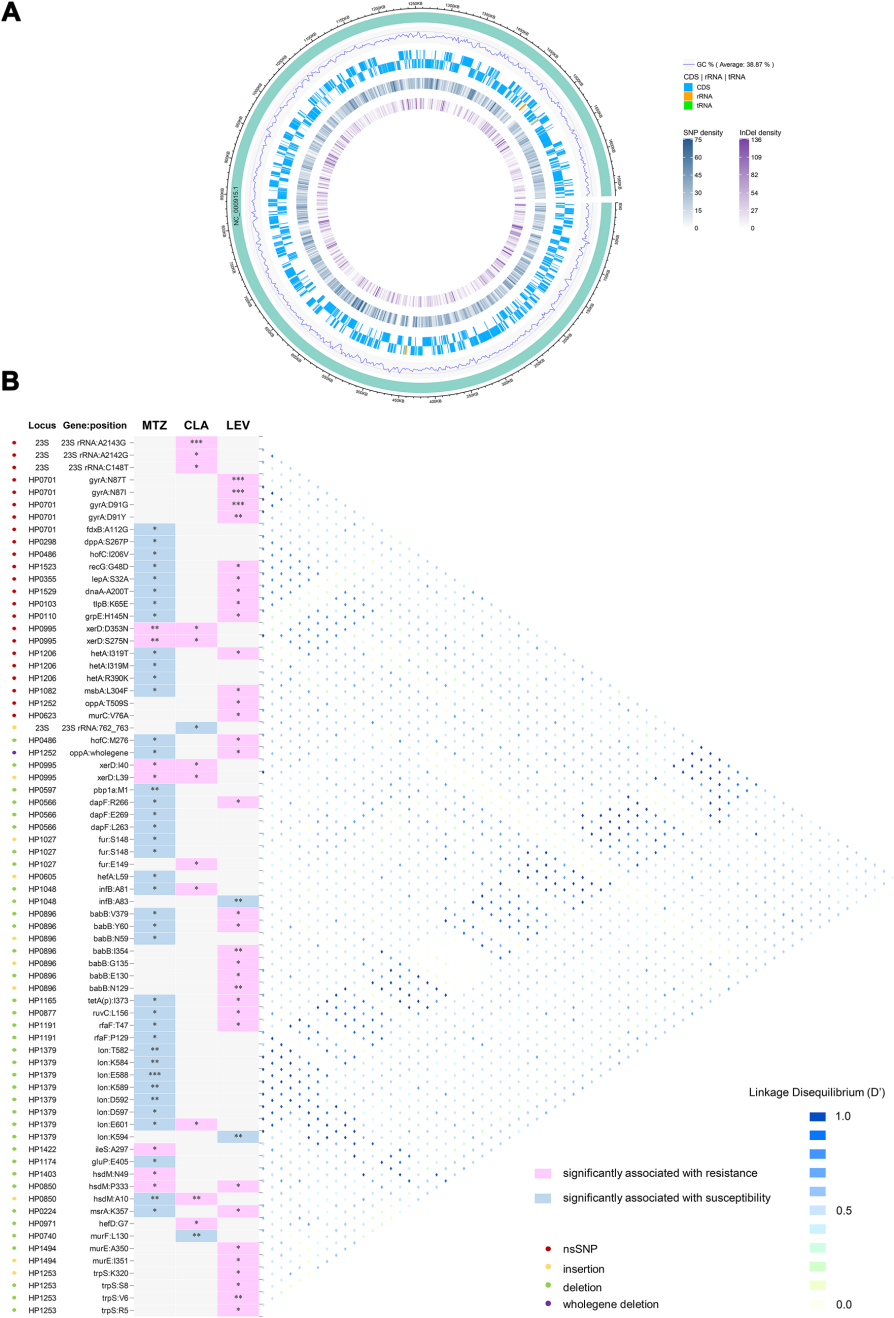
Synoptic representation of the genetic variations in the genome-wide resistance genes and linkage disequilibrium analysis. **A** The densities of total SNPs and Indels identified in the 112 *H. pylori* strains against *H. pylori* 26695 genome are overviewed. The numbers next to the rulers represent the number of SNPs and Indels per unit length of *H. pylori* 26695 genome. **B** The proportions of the strains with the nsSNPs and fsIndels present in the *H. pylori* genome-wide resistant genes in the resistant and susceptible categories of MTZ, CLA and LEV were analyzed, respectively. Only nsSNPs and fsIndels with significantly higher (red cells) or lower (blue cells) frequencies in the resistant categories of MTZ, CLA and LEV were displayed. *P < 0.05, **P < 0.01, ***P < 0.001. Conversely, grey cells indicate the variations not significantly associated with resistance or susceptibility to the antibiotics. Loci, genes, and variations are reported in rows and antibiotics in columns. The nature of the variations is color-coded on the left. The right panel represents the extent of linkage disequilibrium between variations

We called SNPs and Indels within the coding sequences and noncoding rRNAs and tRNAs from the genomes of all the *H. pylori* strains by comparison with the *H. pylori* 26695 reference genome (Fig. 7A), which showed high densities of the variations, which could be as high as 136 for Indel density and 75 for SNP density compared to the reference genome (Fig. 7A). Among these genetic variations, a total of 388 nsSNPs and 1718 fsIndels were identified (Additional file 10: Table S9).

We obtained a list of 80 genes defined as the *H. pylori* genome-wide resistance genes, whose variation, presence, or absence were either known to confer or subsequently found to be associated with *H. pylori* resistance to clinically used antibiotics, not limited to antibiotics with known phenotypes, in this study. To investigate the prevalence of the genetic variations in the resistance genes, we first assayed the variations in the six most common resistance genes, including the 23S rRNA, *gyrA* and *gyrB* genes and the *rdxA*, *frxA,* and *fdxB* genes, in which single-point mutations in specific regions are known to be responsible for CLA, LEV, and MTZ resistance, respectively (Tables 2, 3, 4). Of these SNPs, a subset was significantly associated with AR, including A2143G, A2142G and C148T in the 23S rRNA gene and N87T, N87I, and D91G, D91Y in *gyrA*, as well as I38V in *fdxB*. Additionally, the assay detected four Indels in 23S rRNA, three Indels in *gyrA,* and one in *frxA*. Unexpectedly, the prevalence of one of the Indels (762_763insT) in the 23S rRNA gene was significantly higher in the CLA-S category (53.19%) than in the CLA-R category (Table 2). Furthermore, we overviewed the distribution of the numbers of SNPs and Indels present in the remaining genome-wide resistance genes along with the AR profile of these genes (Additional file 1: Fig. S4). Among the remaining 74 *H. pylori* resistance genes, 174 variations were obtained, corresponding to 54 genes, including 29 genes containing nsSNPs, 36 containing fsIndels, and 10 containing both nsSNPs and fsIndels (Additional file 11: Table S10). Additionally, the maximum number of Indels (ranging from 0 to 15) contained in these genes was up to three times the maximum number of SNPs (ranging from 0 to 3), suggesting that Indel mutations make greater contributions to the high genetic diversity of genome-wide resistance genes than SNP mutations do (Additional file 1: Fig. S4).

**Table 2:** nsSNPs and fsIndels in genes conferring resistance to CLA

Gene	Nucleotide change	No. among CLA-R (n = 18)	No. among CLA-S (n = 94)	*P*
23S rRNA	SNP			
	A2143G	17 (94.44%)	2 (2.13%)	< 0.0001
	A2142G	2 (11.11%)	0 (0.00%)	0.0250
	T2182C	6 (33.33%)	31 (32.98%)	0.9770
	C148T	2 (11.11%)	0 (0.00%)	0.0250
	T1025C	0 (0.00%)	1 (1.06%)	1.0000
	G1027A	14 (77.78%)	52 (55.32%)	0.0760
	Indel			
	761delAA (GAA to G)	16 (88.89%)	84 (89.36%)	1.0000
	762_763insT (A to AT)	5 (27.78%)	50 (53.19%)	0.0482
	764_765insTTT (C to CTTT)	8 (44.44%)	54 (57.45%)	0.3093
	1036_1037insC (A to AC)	18 (100.00%)	94 (100.00%)	1.0000
	1515_1516insT (C to CT)	3 (16.67%)	9 (9.57%)	0.4054
	No change	0 (0.00%)	13 (13.83%)	0.1232

**Table 3:** nsSNPs and fsIndels in genes conferring resistance to LEV

Gene	Sequence change		No. among LEV-R (n = 39)	No. among LEV-S (n = 73)	*P*
	Nucleotide position	Amino acid position			
*gyrA*	SNP				
	C261A	N87T	8 (20.51%)	0 (0.00%)	0.0001
	C261G	N87I	9 (23.08%)	0 (0.00%)	< 0.0001
	G271A	D91G	12 (30.77%)	0 (0.00%)	< 0.0001
	G271T	D91Y	5 (1.67%)	0 (0.00%)	0.0043
	Indel				
	2455_2456insAT (AC to AATC)	T819fs	32 (82.05%)	57 (78.08%)	0.6203
	2460_2463del (CTTCG to C)	S820fs	34 (87.18%)	62 (84.93%)	0.7460
	2472delT (AT to A)	N824fs	38 (97.44%)	66 (90.41%)	0.2577
	No change	No change	1 (2.56%)	7 (9.59%)	0.2577
*gyrB*	SNP				
	C1801T	L601F	37 (94.87%)	73 (100.00%)	0.3423
	No change	No change	2 (5.13%)	0 (0.00%)	0.1192

**Table 4:** nsSNPs and fsIndels in genes conferring resistance to MTZ

Gene	Sequence change		No. among MTZ-R (n = 73)	No. among MTZ-S (n = 39)	*P*
	Nucleotide position	Amino acid position			
*rdxA*	No change	No change	73 (100.00%)	39 (100.00%)	NA
*frxA*	SNP				
	G499A	D167N	4 (5.48%)	4 (10.26%)	0.3497
	T577A	C193S	63 (86.30%)	39 (100.00%)	0.3238
	Indel				
	653dupA (T to TA)	X218delinsX	70 (95.89%)	39 (100.00%)	0.5503
	No change	No change	3 (4.11%)	0 (0.00%)	0.5503
*fdxB*	SNP				
	A112G	I38V	65 (89.04%)	39 (100.00%)	0.0488
	No change	No change	8 (10.96%)	0 (0.00%)	0.0488

ins: insert, del: delete, fs: frameshift, NA: not available

### Analysis of genetic variations in the *H. pylori* genome-wide ARGs

To explore MTZ, CLA, and LEV resistance-related or resistance-induced compensation-related variants, we compared the prevalence of the nsSNPs and fsIndels in the genome-wide ARGs between resistant and susceptible strains. We observed 23 nsSNPs and 48 fsIndels corresponding to 31 genes associated with the resistant or susceptible phenotypes. We found four variations within the integrase/recombinase gene *xerD* showing a significant association with both MTZ and CLA resistance. Both nsSNPs (*xerD*:D353N and *xerD*:S275N) were linked together but exhibited low LD with two *xerD* fsIndels. Similarly, two fsIndels (*xerD*:I40/120_121 delete GG and *xerD*:L39/116_117 insert G) were also linked together (Fig. 7B). A deletion within the site-specific DNA-methyltransferase gene *hsdM* (HP0850) (*hsdM*:P333/998_1002 delete AGCCG) was found to be related to both MTZ and LEV resistance, which were also in high LD with *gyrA*:N87I. Furthermore, we observed 16 variations within 15 genes simultaneously showing an association with MTZ susceptibility and LEV resistance, and many of these genes encoded membrane-related proteins (*hetA*, *msbA*, *hofC*, *oppA*, *tetA(p),* and *babB*) or proteins involved in recombination (*recG* and *ruvC*) (Fig. 7B). Moreover, several deletions within the ATP-dependent serine protease gene *lon* (especially the deletions at T582, K584 and E588) related to MTZ susceptibility exhibited high LD with more than half of the other variations, including the resistance-conferring nsSNPs 23S rRNA:A2143G, 23S rRNA:A2142G, 23S rRNA:C148T, *gyrA*:N87T, and some of the remaining variants, most of which were associated with MTZ resistance or susceptibility (Fig. 7B) (Additional file 12: Table S11).

### Resistance- or susceptibility-associated variations occurred in functionally important domains within the *H. pylori* genome-wide ARGs

**Fig. 8:**
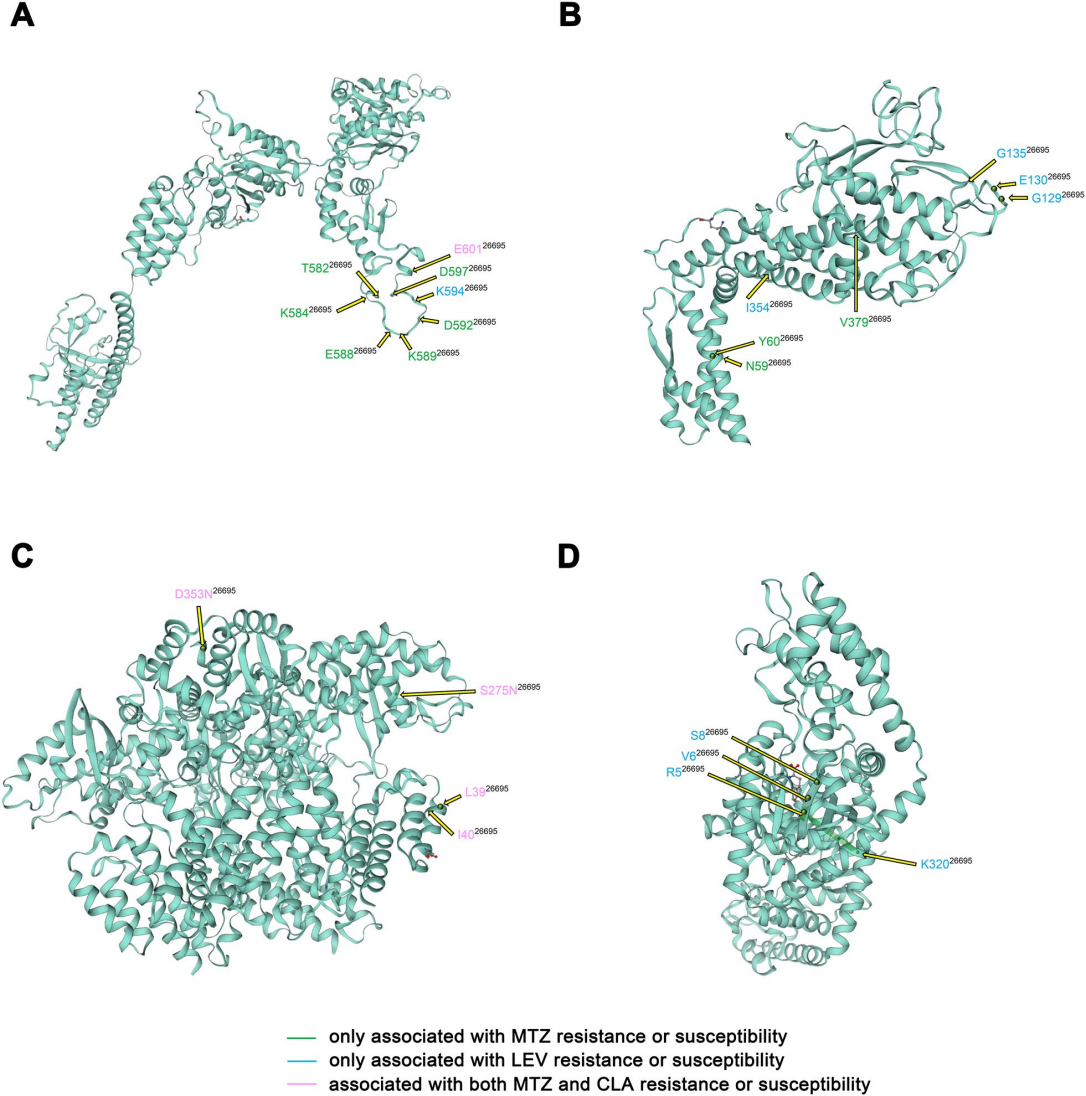
Localization of the nsSNPs and fsIndels in the four proteins with the largest number of resistance-associated variations within the *H. pylori* genome-wide resistant genes. The available crystal structure of each protein was used to indicate the sites with amino acid changes. **A** Lon, **B** BabB, **C** XerD, and **D** TrpS. The color scale represents the corresponding antibiotics of the resistance or susceptibility association

For the purpose of relating the resistance- or susceptibility-related variations to specific protein domains, we further characterized these variations in the genes within the *H. pylori* genome-wide ARGs with corresponding protein structure data. The locations of the nsSNPs and fsIndels in the four proteins (Lon, BabB, XerD, and TrpS) with the largest number of phenotype-associated variations were summarized (Fig. 8), and the functional domains of all the resistance genes with phenotype-associated variations were identified. Eight variations in Lon and three in TrpS were closely located, although they were not in any domains (Fig. 8A and D, Additional file 1: Fig. S5). In BabB, two clusters of physically close variations located at L59, N60, G129, S130, and N135 were all in the sialic acid binding adhesin (SabA) N-terminal extracellular adhesion domain (Fig. 8B, Additional file 1: Fig. S5). Among four variations in XerD, only S275N was located in the phage integrase family domain that enables the protein to be covalently linked to DNA. Of the remaining 27 genes, 14 contained variations totally or partially located in their functional domains as follows: (1) three efflux pump transporter genes: *dppA* (S267P), *hetA* (R390K) and *msbA* (L304F); (2) three lipopolysaccharide (LPS) biosynthesis genes: *murC* (V76A), *murE* (with a deletion at A350 and an insertion at I351), and *rfaF* (with a deletion at P129); (3) two protein translation-related genes: *lepA* (S32A) and *iles* (with a deletion at A297); (4) the metalloregulator gene *fur* (with an insertion at S148 and a deletion at E149); (5) *hofC* (with I206V and a deletion at M276); (6) the stress-response protein coding gene *grpE* (H145N); (7) the chromosomal replication initiator gene *dnaA* (A200T); and (8) the restriction enzyme alleles *hsdM_2* (with a deletion at P333) and *hsdM_3* (with a deletion at N49) (Additional file 1: Fig. S5).

## Discussion

**Fig. 9:**
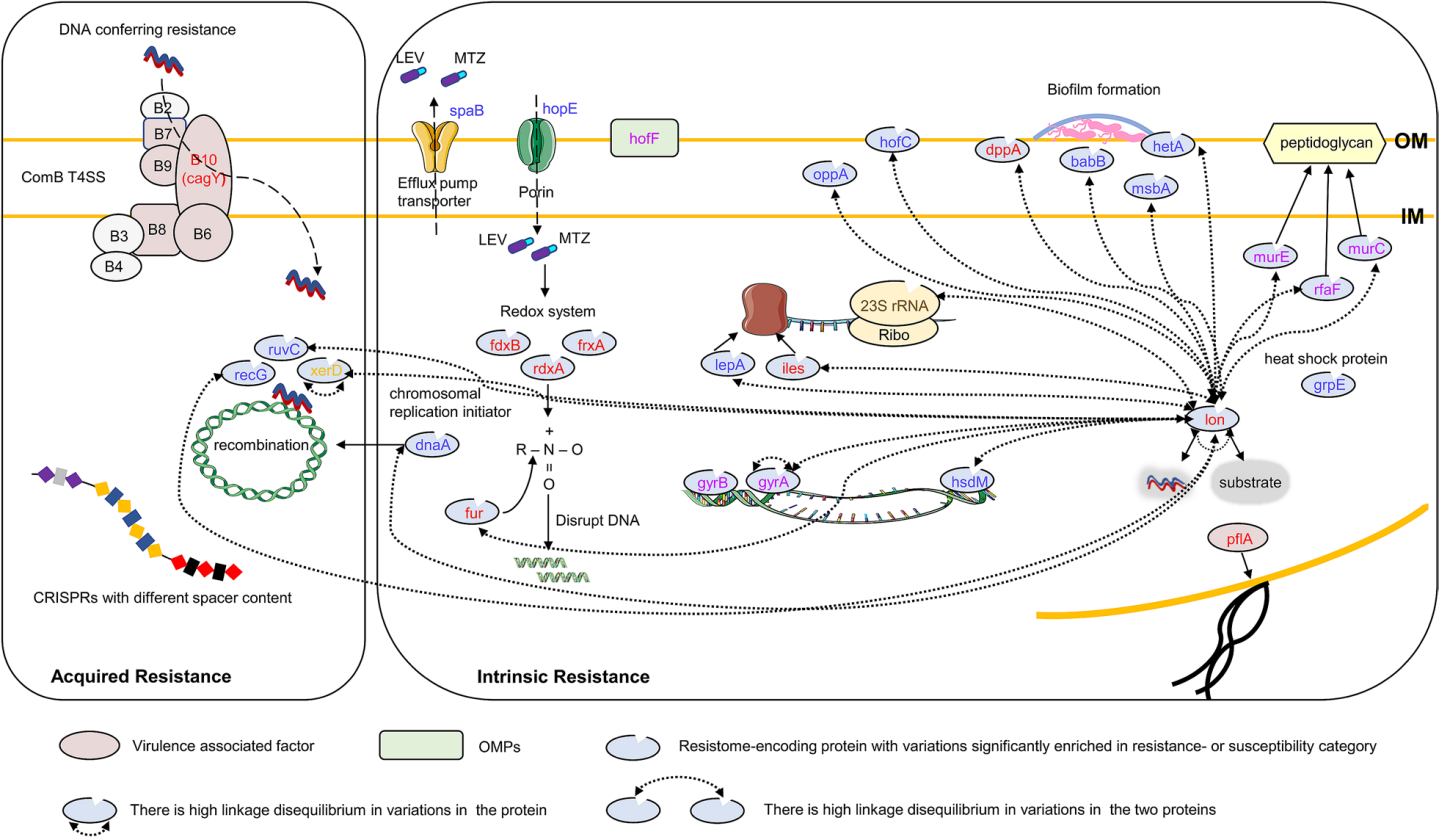
Cartoon model showing the putative genetic characteristics likely related to *H. pylori* resistance- or susceptibility- to MTZ, CLA and LEV at locus- and variant-levels. The intrinsic resistance or resistance-associated compensation related genetic features involves the variations in the genome-wide resistant gene-encoding proteins including antibiotic targets, translation-associated factors, transporters, OMPs, proteins probably related to biofilm formation, and other sporadic factors including lon protease with variations in linkage disequilibrium with many others, as well as the presence or absence of a porin, and an efflux pump transporter. In the underlying acquired resistance related genetic elements, the presence or absence of a virulence-associated factor belonging to ComB T4SS and a certain CRISPRs, as well as variations in several recombination required proteins, were demonstrated. Different colors of the genetic element names denote they are in relation to resistance or susceptibility of different antibiotics. Red (MTZ), gold (CLA), pink (LEV), orange (MTZ and CLA), blue (MTZ and LEV), brown (CLA and LEV)

The emergence and rapid spread of antibiotic-resistant *H. pylori* has become a major obstacle to eradicating the associated infection and has become a great threat to human health. A sharp global decline in the efficacy of the recommended treatment, which used to be 90% effective, has led to an unacceptable threshold (< 80%) for *H. pylori* [4, 51–53]. The ability of *H. pylori* to develop resistance to antibiotics is usually genetically encoded, but only a few of these genetic features have been associated with specific AR. In this study, the clinically used antibiotic (MTZ, CLA, and LEV)-resistance-associated genetic features of 112 *H. pylori*-Shi strains were analysed to explore possible links between specific gene loci and variants with the resistance phenotype (Fig. 9).

Initially, we determined the AR rates of the 112 *H. pylori*-Shi strains with four antibiotics. Our data demonstrated that both mono-resistance (65.2% and 34.8%) and dual-resistance (20.5%) to MTZ and LEV had the highest prevalence compared to the other resistance categories, while the prevalence of resistance to CLA (16.1%) was slightly lower than the resistance rate range of 20% to 50% reported previously in China [7]. Because of the rather low AMX resistance rate of 0% or < 5% worldwide [53] (zero for our 112 *H. pylori*-Shi strains), it was not possible to conduct further association analysis of the resistance mechanism of AMX. In addition, the *H. pylori* strains in this study were isolated from patients with four main gastric diseases, including chronic superficial gastritis, chronic atrophic gastritis, gastric/duodenal ulcer, and gastric cancer, which enabled us to examine potential differences in resistance rates in different specific gastrointestinal pathologies (except gastric cancer, because of insufficient sample number). The negative results implied that the intragastric conditions of patients with different *H. pylori*-linked gastric disorders may have no significant effect on the AR of the pathogen colonizing the gastric mucosa.

Because different phenotypes of *H. pylori* are considered to be associated with unique sets of genetic features [54, 55], we conducted genome-wide gene family analysis to compare the resistant and susceptible categories to obtain unique gene pools and their functional enrichment, which has rarely been investigated in *H. pylori*. Putatively affected by differences in the strain number of each resistant and susceptible category, the level of the unique gene pools of the three antibiotics varied greatly. Nevertheless, nearly half or more than half of them were newly found genes in *H. pylori* with resistance to MTZ (75/183, 40.98%), CLA (5/9, 55.56%), and LEV (13/23, 56.52%), indicating the extensive intraspecies diversity and genetic heterogeneity of *H. pylori* [56].

In our analysis, despite porin gene *hopE* was absent in nearly half of the 112 strains, the gene displayed significant enrichment in the susceptible categories of both MTZ and LEV compared with the resistant population. Similar situations have been reported in a large number of publications showing the loss of porins in clinical strains [57]. It is assumed that bacterial OMPs influence the influx of small-molecule antimicrobials into bacterial cells, and there have been many reports of AR acquired through loss or functional change of porins in a large number of organisms [58]. For example, in *E. coli*, loss of the porins OmpC and OmpF contributes to quinolone resistance [59, 60]. The *A. baumannii* porins OmpA, CarO, Omp33-36, and OprD have been associated with resistance to cephalothin and carbapenem [61–64]. The loss of the OmpK35 and OmpK36 porins in *K. pneumoniae* has been related to resistance to cephalosporins and increased MIC of meropenem [58]. The Hop member HopE, one of the first identified porins in *H. pylori*, is a major nonselective porin that is homologous to the *E. coli* OmpF porin [65, 66]. In our study, the significant loss of *hopE* in the MTZ- and LEV-resistant populations may indicate the underlying effect of *hopE* loss on the development of resistance to the two antibiotics with small molecular weights rather than CLA, which has a large molecular weight.

Regarding the relationship of virulence factors and AR, our analysis suggested the existence of a generally irrelevant relationship between most of the predicted virulence-associated genes and the phenotypes at the “locus” level, including the extensively studied *H. pylori* virulence factors CagA and VacA. This observation is consistent with some reports showing no correlation between AR and the presence of either the *cagA* or *vacA* gene [20, 67], but it is in contrast to many other findings showing that the *cagA*-negative strains and *vacA*-containing strains were associated with CLA resistance [68, 69]. These results remain inconclusive, as they showed a variable pattern, most likely due to low sample numbers in individual studies or variations in geographical regions and evolution. Moreover, we first reported the apparent differences in *cagY* and *pflA* frequencies in MTZ-resistant and MTZ-susceptible *H. pylori*. CagY is an orthologue of VirB10 and is an essential gene in the ComB type IV secretion system (T4SS) [70]. Rather than the canonical type IV (pseudo) pilus used by other Gram-negative bacteria, *H. pylori* employs the ComB system for initial DNA uptake during natural transformation [71]. Thus, the significantly higher frequency of *cagY* in the MTZ resistance category suggests that the functional ComB system with intact CagY enables the entry of resistance-associated DNA via transformation, which promotes the development of MTZ resistance. Further research is needed to identify and better understand the specific mechanisms behind this observation.

Previous reports have demonstrated that the AcrAB-TolC efflux pump belonging to the RND family encoding hefABC, hefDEF, and hefGHI is the most important efflux system in *H. pylori,* mainly mediating resistance to several antibiotics, such as MTZ and CLA, under laboratory conditions [11, 15]. It has been reported that AcrAB-TolC is associated with the development of resistance to CLA represented by the reduced lowest inhibitory concentration observed when using RND pump inhibitors [72]. Moreover, the RND pumps may contribute to MTZ resistance in *H. pylori* because the expression of the TolC homologous genes was elevated in MTZ-resistant isolates [73]. However, our analysis showed that the frequencies of the RND family efflux pump genes in the resistant and susceptible categories were not significantly different. We also reported here that the ABC family member spaB showed a difference in frequency between the resistant and susceptible categories of both MTZ and LEV, which is consistent with a previous report by Yang et al. from China showing that the expression level of *spaB* in MDR strains was significantly higher than that in susceptible strains and that the sensitivity of *spaB*-knockout *H. pylori* to four antibiotics (LEV, CLA, chloramphenicol and rifampicin) was significantly higher than that of wild-type strains (reference not provided). Similar studies on spaB have not been reported in the rest of the world. Whether the transporter is involved in *H. pylori* resistance remains to be determined.

Studies investigating the characteristics of CRISPR-Cas systems in relation to AR in clinical *H. pylori* strains are lacking. These are significant gaps in knowledge because the CRISPR-Cas system is hypothesized to be a natural impediment to horizontal gene transfer (HGT) in bacteria [74] and because HGT disseminates AR-associated genetic elements among competent pathogens in vivo [75], and it is essential to identify strategies to reduce the spread of these elements. Nevertheless, our analysis failed to predict any acquired ARGs where resistance was conferred by a complete gene. Given the high rate of the naturally competent transformation of *H. pylori* [76], it is plausible that the transfer of single-nucleotide mutations conferring AR rather than complete ARGs plays a prominent role in the horizontally acquired resistance of *H. pylori*, which has been supported by a series of reports [26, 71, 77]. Furthermore, we found that LEV resistance and possession of complete CRISPR loci were inversely related. Similar associations showing that the CRISPR system was more likely to be found in antimicrobial-susceptible species such as *E. coli* and *Enterococci* have also been reported [74, 78, 79]. Additionally, our correlation of a different number of spacers with resistance or susceptibility to LEV or MTZ, as well as the interesting finding of a CRISPR combination and a DR sequence occurring exclusively in MTZ-resistant strains, provided an initial foundation for potentially utilizing the spacer content or DR sequence to rapidly determine the phenotype of *H. pylori* strains.

In the phylogenetic analysis, we noticed that a subset of 8 isolates in a subclade showed high separation, indicating that these strains had considerable differences in genetic similarity and relatively distant genetic relationships compared with the genomes of other strains.

Similar to other bacteria with small genomes, the responses of *H. pylori* to environmental pressure rely mainly on variations rather than a complex regulatory network [80]. Next-generation sequencing is a powerful tool to compare whole genomes and analyse the variations found within AR-associated genes. When compiling the *H. pylori* genome-wide resistance gene list based on databases, no β-lactamase genes were found in the 112 *H. pylori* strains, which was consistent with a previous report showing that the ß-lactamase gene could be detected in AMX-resistant *H. pylori* but not in AMX-susceptible strains [81]. Regarding the antibiotic target genes, not only the well-known mutations in *gyrA* (N87T/I and D91G/Y) for LEV resistance and mutations in 23S rRNA (A2143G and A2142G) for CLA resistance but also several other single-nucleotide mutations and Indels associated with AR or antibiotic susceptibility were detected, and the latter could be compensatory mutations to mitigate the deleterious effects of the specific functions compromised by the resistance mutations [82]. In the other variations in the genome-wide resistance genes associated with AR or antibiotic susceptibility, we noticed MTZ susceptibility-associated variations in *fur*. MTZ belonging to the nitroimidazole class is a prodrug that is activated by nitroreductases, and the reduction products can disrupt DNA. It was suggested that the resistance stemming from inactive nitroreductases can be eliminated by mutations in the fur protein [83]. The LD analysis among these variations revealed several notable deletion mutations in *lon* that were found to be in high LD with more than half of the other variations, including the resistance-conferring nsSNPs. Given that the *lon*-encoded protease mediates the selective degradation of mutants and abnormal proteins [84, 85], we speculated that deletion mutations at specific sites of *lon* indirectly participate in the resistance mechanisms of *H. pylori* by influencing the effect of *lon* in degrading mutants or proteins encoded by mutants potentially related to the AR or antibiotic susceptibility of *H. pylori*, which may explain their linked co-occurrence to a certain extent. Further structure and functional domain analysis of genome-wide resistance genes containing resistance- or susceptibility-associated variations better indicated their probable involvement in the regulation of AR. For example, five Indels were present in the SabA N-terminal extracellular adhesion domain of BabB. This domain is conserved among SabA orthologues and BabA and is known to function as a sugar-binding adhesion domain with conserved disulfide bonds [86]. Smiley et al. previously reported that the transmembrane protein BabB was upregulated in CLA-resistant *H. pylori* [12]. The other functional domains in the genome-wide resistance genes containing variations included regions important for efflux (dppA, hetA and msbA), LPS biosynthesis (murC, murE and rfaF) and stress response (grpE). It has been reported that adhesins (especially OMPs from the Hop and Hom families) [87–89], LPS [87, 89, 90], efflux pumps [14, 87, 91] and proteins regulating a stress response [87, 88] are all associated with the biofilm formation of *H. pylori*. Thus, it may be the case that biofilm formation influenced by these genetic mutants is an important mechanism in the AR of *H. pylori*, which needs to be substantiated via extensive experiments.

## Conclusion

Our study demonstrated that *H. pylori* strains isolated from Shanghai exhibited multidrug resistance and correctly identified the well-known mutations conferring intrinsic resistance to MTZ, CLA and LEV. Furthermore, we found that AR phenotypes were associated with a number of novel locus- and variant-level genetic elements. The underlying co-occurrence relationships among them were also revealed. These findings provide a data basis for further resistance-associated research. Moreover, thorough detection and characterization of AR in *H. pylori* strains is necessary to develop efficacious treatment regimens. Continued monitoring of AR using susceptibility assays and genotyping is warranted in Shanghai.

## Supplementary Information

**Additional file 1: Figure S1.** Colony morphology of sub-cultured *H. pylori *strains on the selective plate. **Figure S2. **Local quality estimates and comparison plots of the established protein structure models. The Local Qualit Estimate shows, for each residue of the model (reported on the x-axis), the expected similarity to the native structure (y-axis). Typically, residues showing a score below 0.6 are expected to be of low quality. Different model chains are shown in different colous. **(A)** Lon, **(C)** BabB, **(E)** XerD, **(G)** TrpS. Generally, model quality scores of individual models are related to scores obtained for experimental structures of similar size. In the Comparison plot, the x-axis shows protein length (number of residues). The y-axis is the normalized QMEAN score. Every dot represents one experimental protein structure. Black dots are experimental structures with a normalized QMEAN score within 1 standard deviation of the mean (|Z-score| between 0 and 1), experimental structures with a |Z-score| between 1 and 2 are grey. Experimental structure that are even further from the mean are light grey. The actual model is represented as a red star. **(B)** Lon, **(D)** BabB,** (F)** XerD, **(H)** TrpS. **Figure S3. **The structural analysis of two other CRISPRs containing the DR exclusively presenting in nine MTZ-R strains.** (A, B)** DRs are shown as red diamonds and spacers are shown as green and blue rectangles in each CRISPR. The base sequences are shown below the CRISPR array. The DRs and spacers in the colored characters correspond with the colors of the respective diamonds and rectangles. The DR sequence exclusively presenting in nine MTZ-R strains is in box. **Figure S4. **Heatmap of the numbers of the variations presented in the genes of the *H. pylori *resistome (in addition to 23S rRNA, *gyrA*, *gyrB*, *rdxA*, *frxA* and *fdxB* genes) in the 112 strains. Heatmaps showing the distribution of the numbers of the nsSNPs **(A)** and the fsIndels **(B)** presented in the remaining genes of the *H. pylori *resistome in the 112 isolates categorized by phenotypes of three antibiotics involved in this study. The genes and the corresponding loci as well as the antibiotic resistant profile of the genes are listed on the right. Gens within the resistome with no SNPs or Indels are not included in the heatmaps. The different numbers of the nsSNPs or the fsIndels are represented by different colors displayed on the right panel. **Figure S5. **Functionally important domains of genes containing the resistance- or susceptibility-associated variations within the *H. pylori *resistome. The length of each bar indicates the size of the gene. The regions with different colors represent the corresponding functional domains. The red indicator line represents the variation site, with the position information labeld above it.

**Additional file 2: Table S1.** Demographics, clinical characteristics and antibiotic resistance phenotypes.

**Additional file 3: Table S2.** General genomic features for the 112 H. pylori isolates.

**Additional file 4: Table S3.** The databases- and literature- supported H. pylori resistome.

**Additional file 5: Table S4.** Source data of the inclusion situation of the resistanceunique genes in the NCBI-NR, SwissProt and COG databases.

**Additional file 6: Table S5.** List of unique genes of MTZ-R, CLA-R and LEV-R categories.

**Additional file 7: Table S6.** Distribution of the outer membrane protein family genes in the resistant and sensitive categories.

**Additional file 8: Table S7.** Distribution of the predicted virulence-associated genes in the resistant and sensitive categories.

**Additional file 9: Table S8.** Source data of the CRISPRs and DR sequences in the 112 *H. pylori* strains.

**Additional file 10: Table S9.** Source data of the nsSNPs and fsIndels identified in the 112 *H. pylori* strains.

**Additional file 11: Table S10.** The 173 variations found in the 54 genes within the H. pylori resistome.

**Additional file 12: Table S11.** Source data of the LD analysis.

## Data Availability

The Whole Genome Shotgun project of the 112 *H. pylori*-Shi strains has been deposited at DDBJ/ENA/GenBank (BioProject number PRJNA690879) under the accessions shown in Additional file 3: Table S2. Additional raw data is available in Additional files 4–12: Table S3–S11.
