# Supplementary material for: Antimicrobial resistance patterns and genetic elements associated with the antibiotic resistance of Helicobacter pylori strains from Shanghai
Source: Gut Pathog. 2022 Mar 30;14:14. doi: 10.1186/s13099-022-00488-y (PMC8966258; doi:10.1186/s13099-022-00488-y)
Supplement: Supplementary file 1 — Additional file 1: Figure S1. Colony morphology of sub-cultured H. pylori strains on the selective plate. Figure S2. Local quality estimates and comparison plots of the established protein structure models. The Local Qualit Estimate shows, for each residue of the model (reported on the x-axis), the expected similarity to the native structure (y-axis). Typically, residues showing a score below 0.6 are expected to be of low quality. Different model chains are shown in different colous. (A) Lon, (C) BabB, (E) XerD, (G) TrpS. Generally, model quality scores of individual models are related to scores obtained for experimental structures of similar size. In the Comparison plot, the x-axis shows protein length (number of residues). The y-axis is the normalized QMEAN score. Every dot represents one experimental protein structure. Black dots are experimental structures with a normalized QMEAN score within 1 standard deviation of the mean (|Z-score| between 0 and 1), experimental structures with a |Z-score| between 1 and 2 are grey. Experimental structure that are even further from the mean are light grey. The actual model is represented as a red star. (B) Lon, (D) BabB, (F) XerD, (H) TrpS. Figure S3. The structural analysis of two other CRISPRs containing the DR exclusively presenting in nine MTZ-R strains. (A, B) DRs are shown as red diamonds and spacers are shown as green and blue rectangles in each CRISPR. The base sequences are shown below the CRISPR array. The DRs and spacers in the colored characters correspond with the colors of the respective diamonds and rectangles. The DR sequence exclusively presenting in nine MTZ-R strains is in box. Figure S4. Heatmap of the numbers of the variations presented in the genes of the H. pylori resistome (in addition to 23S rRNA, gyrA, gyrB, rdxA, frxA and fdxB genes) in the 112 strains. Heatmaps showing the distribution of the numbers of the nsSNPs (A) and the fsIndels (B) presented in the remaining genes of the H. pylori resistome [file 13099_2022_488_MOESM1_ESM.docx]

**Additional file 1: Figures S1–S5**

**Antimicrobial resistance patterns and genetic elements** **associated with the antibiotic resistance of *Helicobacter pylori* strains from Shanghai**

**Yixin Liu^1,2,3^**^†^**, Su Wang^1^**^†^**, Feng Yang^1,2,3^, Wenjing Chi^1^, Li Ding^1^, Tao Liu^1^, Feng Zhu^1^, Danian Ji^4^, Jun Zhou^4^, Yi Fang^1^, Jinghao Zhang^1^, Ping Xiang^4^, Yanmei Zhang^1,2,3*^, Hu Zhao^1,2,3*^**

^1^ Department of Laboratory Medicine, Huadong Hospital, Fudan University, Shanghai, China

^2^ Shanghai Key Laboratory of Clinical Geriatric Medicine, Shanghai, China

^3^ Research Center on Aging and Medicine, Fudan University, Shanghai, China

^4^ Department of Endoscopy, Huadong Hospital, Fudan University, Shanghai, China

^*^Correspondence:
Yanmei Zhang: [15618653286@163.com](mailto:15618653286@163.com); Hu Zhao: [hubertzhao@163.com](mailto:hubertzhao@163.com)

†These authors have contributed equally to this work.


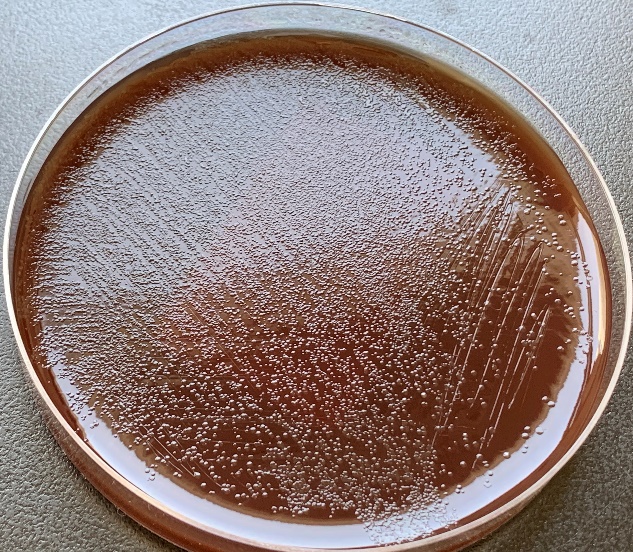


**Figure S1 Colony morphology of sub-cultured *H. pylori* strains on the selective plate.**


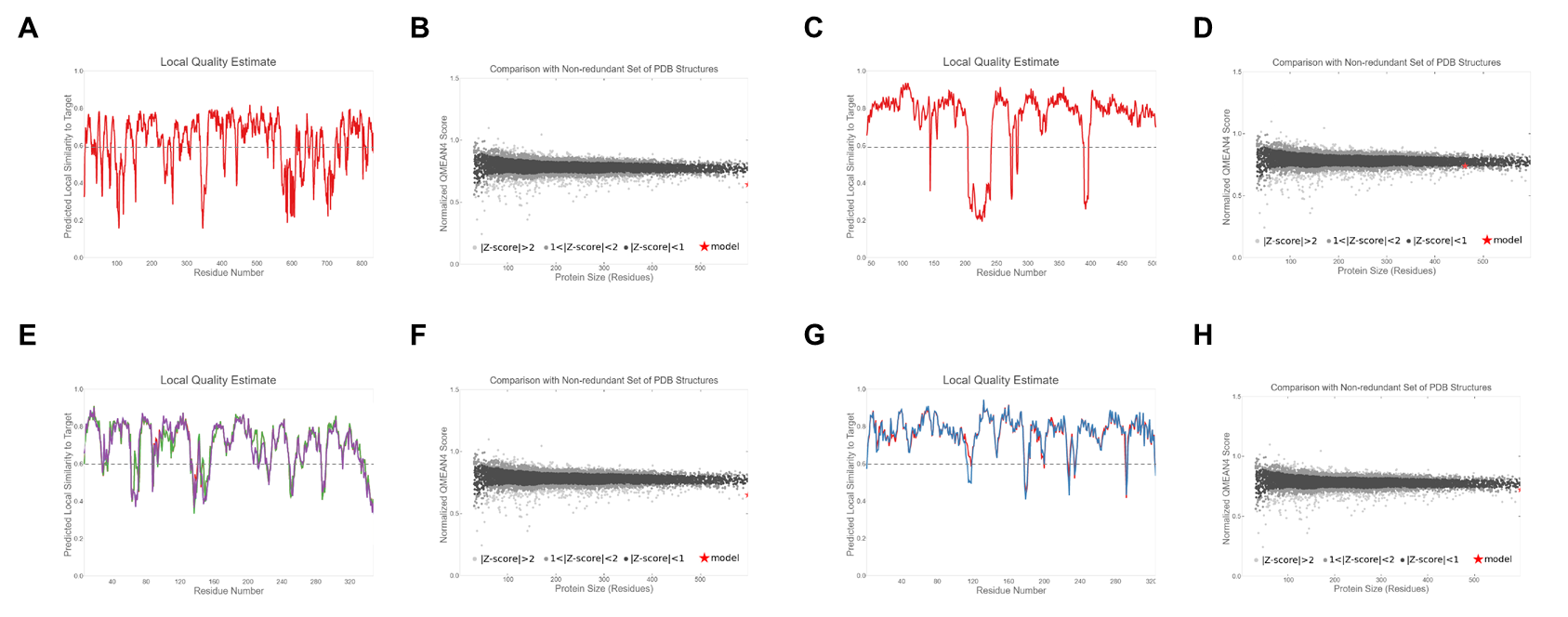


**Figure S2 Local quality estimates and comparison plots of the established protein structure models.** The Local Qualit Estimate shows, for each residue of the model (reported on the x-axis), the expected similarity to the native structure (y-axis). Typically, residues showing a score below 0.6 are expected to be of low quality. Different model chains are shown in different colous. **(A)** Lon, **(C)** BabB, **(E)** XerD, **(G)** TrpS. Generally, model quality scores of individual models are related to scores obtained for experimental structures of similar size. In the Comparison plot, the x-axis shows protein length (number of residues). The y-axis is the normalized QMEAN score. Every dot represents one experimental protein structure. Black dots are experimental structures with a normalized QMEAN score within 1 standard deviation of the mean (|Z-score| between 0 and 1), experimental structures with a |Z-score| between 1 and 2 are grey. Experimental structure that are even further from the mean are light grey. The actual model is represented as a red star. **(B)** Lon, **(D)** BabB, **(F)** XerD, **(H)** TrpS.


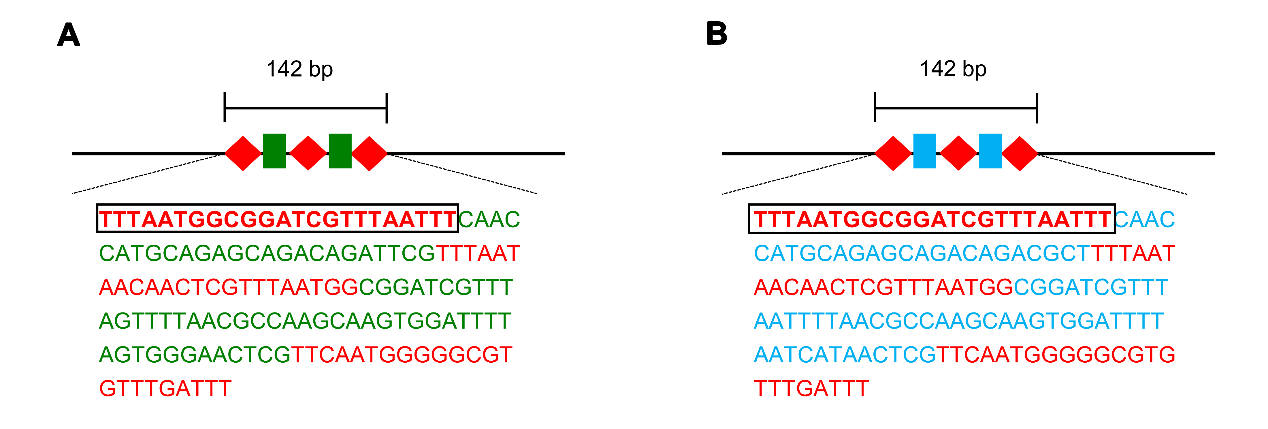


**Figure S3 The structural analysis of two other CRISPRs containing the DR exclusively presenting in nine MTZ-R strains. (A, B)** DRs are shown as red diamonds and spacers are shown as green and blue rectangles in each CRISPR. The base sequences are shown below the CRISPR array. The DRs and spacers in the colored characters correspond with the colors of the respective diamonds and rectangles. The DR sequence exclusively presenting in nine MTZ-R strains is in box.


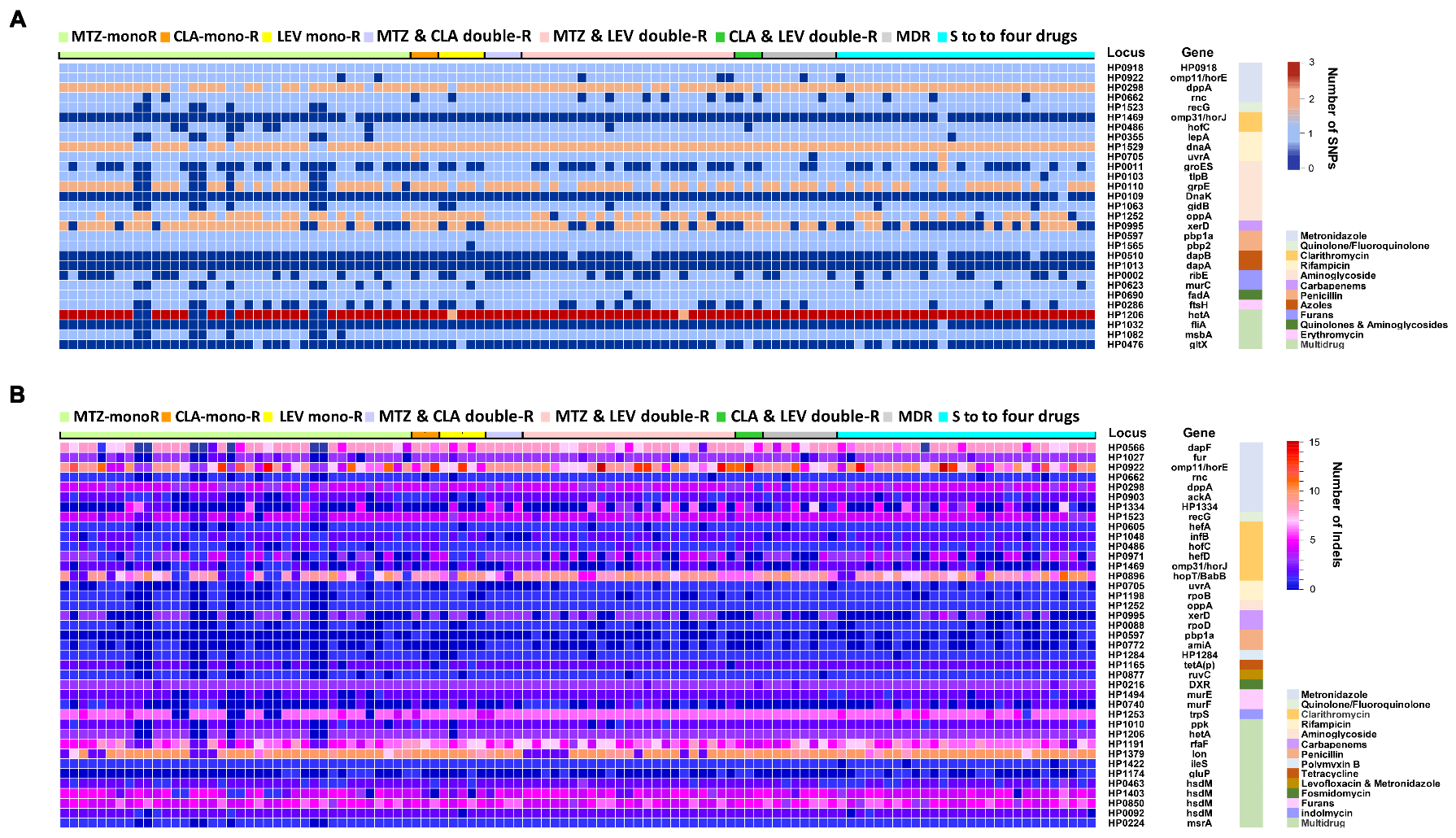


**Figure S4 Heatmap of the numbers of the variations presented in the genes of the *H. pylori* resistome (in addition to 23S rRNA, *gyrA*, *gyrB*, *rdxA*, *frxA* and *fdxB* genes) in the 112 strains.** Heatmaps showing the distribution of the numbers of the nsSNPs **(A)** and the fsIndels **(B)** presented in the remaining genes of the *H. pylori* resistome in the 112 isolates categorized by phenotypes of three antibiotics involved in this study. The genes and the corresponding loci as well as the antibiotic resistant profile of the genes are listed on the right. Gens within the resistome with no SNPs or Indels are not included in the heatmaps. The different numbers of the nsSNPs or the fsIndels are represented by different colors displayed on the right panel.


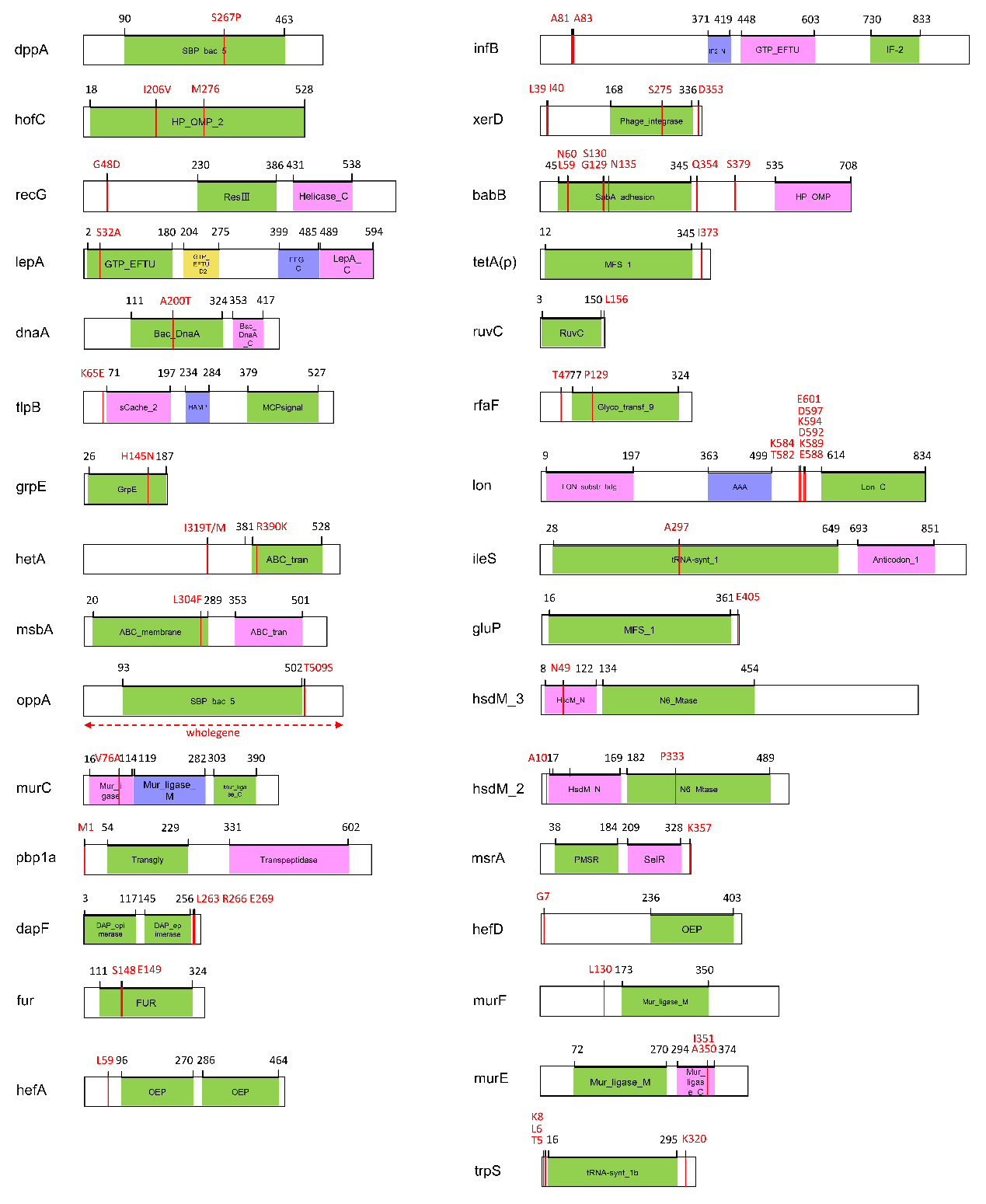


**Figure S5 Functionally important domains of genes containing the resistance- or susceptibility-associated variations within the *H. pylori* resistome.** The length of each bar indicates the size of the gene. The regions with different colors represent the corresponding functional domains. The red indicator line represents the variation site, with the position information labeld above it.
